# Role of hepcidin in oxidative stress and cell death of cultured mouse renal collecting duct cells: protection against iron and sensitization to cadmium

**DOI:** 10.1007/s00204-021-03106-z

**Published:** 2021-06-28

**Authors:** Stephanie Probst, Johannes Fels, Bettina Scharner, Natascha A. Wolff, Eleni Roussa, Rachel P. L. van Swelm, Wing-Kee Lee, Frank Thévenod

**Affiliations:** 1 Faculty of Health, Institute of Physiology, Pathophysiology and Toxicology and ZBAF (Centre for Biomedical Education and Research), School of Medicine, Witten/Herdecke University, Stockumer Str 12 (Thyssenhaus), 58453 Witten, Germany; 2 Department of Molecular Embryology, Faculty of Medicine, Institute of Anatomy and Cell Biology, University of Freiburg, Albertstr. 17, 79104 Freiburg, Germany; 3 Department of Laboratory Medicine, Radboud Institute for Molecular Life Sciences, Radboud University Medical Center, Geert Grooteplein 10, 6525 GA Nijmegen, The Netherlands; 4 AG Physiology and Pathophysiology of Cells and Membranes, Medical School OWL, Bielefeld University, Morgenbreede 1, 33615 Bielefeld, Germany

**Keywords:** DFO, Metallothionein, Ca_V_3.1, DMT1

## Abstract

The liver hormone hepcidin regulates systemic iron homeostasis. Hepcidin is also expressed by the kidney, but exclusively in distal nephron segments. Several studies suggest hepcidin protects against kidney damage involving Fe^2+^ overload. The nephrotoxic non-essential metal ion Cd^2+^ can displace Fe^2+^ from cellular biomolecules, causing oxidative stress and cell death. The role of hepcidin in Fe^2+^ and Cd^2+^ toxicity was assessed in mouse renal cortical [mCCD(cl.1)] and inner medullary [mIMCD_3_] collecting duct cell lines. Cells were exposed to equipotent Cd^2+^ (0.5–5 μmol/l) and/or Fe^2+^ (50–100 μmol/l) for 4–24 h. Hepcidin (*Hamp1*) was transiently silenced by RNAi or overexpressed by plasmid transfection. Hepcidin or catalase expression were evaluated by RT-PCR, qPCR, immunoblotting or immunofluorescence microscopy, and cell fate by MTT, apoptosis and necrosis assays. Reactive oxygen species (ROS) were detected using CellROX™ Green and catalase activity by fluorometry. Hepcidin upregulation protected against Fe^2+^-induced mIMCD_3_ cell death by increasing catalase activity and reducing ROS, but exacerbated Cd^2+^-induced catalase dysfunction, increasing ROS and cell death. Opposite effects were observed with *Hamp1* siRNA. Similar to *Hamp1* silencing, increased intracellular Fe^2+^ prevented Cd^2+^ damage, ROS formation and catalase disruption whereas chelation of intracellular Fe^2+^ with desferrioxamine augmented Cd^2+^ damage, corresponding to hepcidin upregulation. Comparable effects were observed in mCCD(cl.1) cells, indicating equivalent functions of renal hepcidin in different collecting duct segments. In conclusion, hepcidin likely binds Fe^2+^, but not Cd^2+^. Because Fe^2+^ and Cd^2+^ compete for functional binding sites in proteins, hepcidin affects their free metal ion pools and differentially impacts downstream processes and cell fate.

**Supplementary Information:**

The online version contains supplementary material available at 10.1007/s00204-021-03106-z.

## Introduction

Synthesis and secretion of the systemic iron-regulatory hormone hepcidin (rodent gene name *Hamp1*) by the liver is regulated by iron stores within macrophages, inflammation, hypoxia, and erythropoiesis (reviewed in Ganz and Nemeth 2012). Binding of circulating hepcidin to its target protein ferroportin-1 on enterocytes and macrophages leads to internalization and degradation of the hepcidin-ferroportin-1 complex by ubiquitination, thereby inhibiting the efflux of iron from intestinal enterocytes into the blood as well as preventing the release of stored iron from macrophages (Nemeth et al. 2004). Together, these effects result in a decrease in circulating iron levels.

The kidney has recently emerged as an organ with a significant role in systemic iron homeostasis (Thévenod and Wolff 2016). Substantial amounts of iron are filtered by the kidney and have to be reabsorbed to prevent iron deficiency. Accordingly, iron transporters and receptors for protein-bound iron are expressed in the nephron (reviewed in Martines et al. 2013; Smith and Thévenod 2009; Thévenod and Wolff 2016). Systemic iron overload induced by genetic diseases, such as hereditary haemochromatosis or β-thalassemia major result in renal iron deposition and kidney injury involving oxidative stress induced by the Fenton metal ion Fe^2+^ (Halliwell and Gutteridge 2015), various forms of cell death or inflammation (reviewed in van Swelm et al. 2020). It is intriguing that hepcidin is also locally expressed in various organs, including the kidney where it is found in the distal nephron, but not in the proximal tubule (PT) (Kulaksiz et al. 2005). Although several studies indicate that hepcidin protects against certain forms of kidney injury caused by oxidative stress (Scindia et al. 2015; van Swelm et al. 2016, 2018), and possibly interferes with cell death signaling or chelates Fe^2+^ (Farnaud et al. 2006, 2008; Gerardi et al. 2005), the function of hepcidin locally expressed in the distal nephron is not understood.

Cadmium (Cd^2+^) is a non-essential transition metal, which is taken up from the environment into the body through pulmonary and enteral pathways (Jarup and Akesson 2009). The kidney PT is a major target of Cd^2+^ toxicity by chronic (low) Cd^2+^ exposure (reviewed in Thévenod 2003, 2009). Renal dysfunction develops in up to 7% of the general population, and in its most severe form displays major features of renal *Fanconi* syndrome (Johri et al. 2010). Although Cd^2+^ is not a Fenton metal ion, it also increases cellular reactive oxygen species (ROS) formation, e.g., by disrupting ROS metabolizing enzymes (reviewed in Cuypers et al. 2010), which alters their anti-oxidative redox signature and leads to apoptotic and/or necrotic cell death, as happens in PT cells (Nair et al. 2015). The renal medulla also accumulates significant amounts of Cd^2+^ in humans and concentrations of Cd^2+^ can reach ~ 50% of the levels found in the cortex (Nagamine et al. 2007; Torra et al. 1994; Wang et al. 2009; Yoshida et al. 1998), although nephrotoxicity is less apparent (Johri et al. 2010). Cd^2+^ has similar bioinorganic chemical characteristics as Fe^2+^, and it can displace Fe^2+^ from its physiological binding sites (including Fe^2+^ transporters) to disrupt cellular functions and cause damage (reviewed in Moulis 2010). Indeed, the distal nephron expresses a variety of Cd^2+^ entry pathways, including iron response element (IRE)-regulated Fe^2+^ transporters (reviewed in Smith and Thévenod 2009; Thévenod and Wolff 2016).

Consequently, we assessed roles of hepcidin in Fe^2+^- and Cd^2+^-induced ROS formation and cell injury in the mouse kidney cortical and inner medullary collecting duct (CD) cell lines mCCD(cl.1) and mIMCD_3_.

## Materials and methods

### Experimental solutions

A CdCl_2_ stock solution (Merck; cat. # 2011; 2.5 mM dissolved in ddH_2_O) was used throughout the study and diluted 1:500–1:1000 in serum-free medium. Iron salts and Na^+^-ascorbate stock solutions were freshly prepared for each experiment. Stock solutions were as follows: FeSO_4_ (Sigma-Aldrich; cat. # F8048) or FeCl_3_ (Sigma-Aldrich; cat. # 157740), 5 mM in ddH_2_O or in 500 mM Na^+^-ascorbate, pH 7.4 where ascorbic acid (C_6_H_8_O_6_) (Sigma-Aldrich; cat. # A5960) was dissolved in ddH_2_O, pH titrated to 7.4 with 1 M NaOH and final volume adjusted to obtain a 500 mM stock solution. Stock solutions of iron salts and Na^+^-ascorbate were diluted 1:50 or 1:100 in serum-free medium for individual experiments. Stock solutions of 10 mmol/l desferrioxamine mesylate salt (Sigma-Aldrich; cat. # D9533) dissolved in phosphate-buffered saline (PBS) were freshly prepared prior to use.

### Cell culture

The immortalized mouse kidney medulla CD cell line mIMCD_3_, was obtained from ATCC (cat. # ATCC CRL-2123). The mCCD(cl.1) cell line clone was originally derived from primary cultures of cortical CD microdissected from mouse kidney (Gaeggeler et al. 2005) and was obtained from Dr. Edith Hummler (University of Lausanne, CH). WKPT-0293 Cl.2 cells, an immortalized cell line from the S1 segment of rat PT, was obtained from Dr. Ulrich Hopfer (Case Western, Reserve University, Cleveland, OH, USA) (Woost et al. 1996). mIMCD_3_ cells were cultured in Dulbecco’s modified Eagle’s medium (DMEM)/nutrient mixture F-12 (1:1) (GibCo; cat. # 31330) supplemented with 10% fetal bovine serum (FBS) (Gibco; cat. # 10270-106), 50 U/ml penicillin, and 50 μg/ml streptomycin (Gibco; cat. #15140-122). mCCD(cl.1) cells were cultured in DMEM/F-12 (1:1) supplemented with 5% FBS, 100 U/ml penicillin, and 100 μg/ml streptomycin, 0.9 μmol/l insulin (Sigma–Aldrich; cat. # I1882), 5 μg/ml apo-transferrin (Sigma-Aldrich; cat. # T2252), 10 ng/ml EGF (Sigma-Aldrich; cat. # E9644), 1 nM T3 (Sigma-Aldrich; cat. # T6397), and 50 nM dexamethasone (Sigma-Aldrich; cat. # D4902) (Fila et al. 2011). WKPT-0293 Cl.2 cells were cultured in medium essentially as previously described (Lee et al. 2005). All cell lines were used at passage numbers 20–40 and cultured in 25 or 75 cm^2^ standard tissue culture flasks (Sarstedt) at 37°C in a humidified 5% CO_2_ atmosphere and passaged twice a week upon reaching ~ 80–90% confluency.

### Animals

Six- to eight-week-old C57BL/6 mice of either sex (Charles River Laboratories) were provided food and water ad libitum and maintained through a 12–12 h light–dark cycle in a climate-controlled environment. Animal handling and euthanasia were performed in accordance with the recommendations of German Ethical Guidelines for Laboratory Animals and the European Directive on the Protection of Animals used for Scientific Purposes (2010/63/EU). Protocols were approved by the Governmental Animal Ethics Committee of North-Rhine-Westphalia, Germany (Landrat Ennepe-Ruhr-Kreis, file number 32/7 from January 20, 2014) and the Institutional Animal Care and Use Committee of the University of Freiburg and the ethics committee of the City of Freiburg (authorization: X18/08C). All efforts were made to minimize the number of animals used and their suffering. Mice were anaesthetized with CO_2_ and/or sacrificed by cervical dislocation, and kidneys were immediately excised. For qPCR and immunoblotting, kidney cortex was grossly separated from medulla by surgery using a scalpel and razor blades.

### Transient transfections

Transient transfection of *Hamp1* siRNA or expression plasmid were performed according to manufacturers’ protocols. In brief, 100 nM mouse *Hamp1* (Hamp1si) (Sigma-Aldrich; cat # EMU174481) or 100 nM control siRNA (ctrlsi) (Eurogentec, cat. # SR-CL000-005) was mixed with Lipofectamine RNAiMAX (Thermo Fisher Scientific) in Opti-MEM I (Gibco) and incubated for 5 min at room temperature (RT). For PCR analyses, 10–100 nM siRNA was used. For plasmid transfection, 0.5–1.25 µg mouse *Hamp1* plasmid (Origene, cat. # MC211974) or control plasmid were mixed with Lipofectamine 2000 (Thermo Fisher Scientific) in Opti-MEM I and incubated for 10 min at RT prior to transfection in 0.5–1 ml culture medium. For *Hamp1* gene silencing or overexpression, cells were exposed for 6 h to siRNA or plasmid solutions in fresh serum-free standard medium without antibiotics. Medium was then replaced by siRNA- and serum-containing or serum-free medium with antibiotics and cultured for up to additional 18 h (24 h in total with or without experimental treatments). For hepcidin immunofluorescence staining in mCCD(cl.1), a different protocol was used: after transfection with siRNA for 6 h in serum-free medium, cells were cultured for additional 18 h in standard serum-containing medium with antibiotics before exposure to serum-free medium for 6 h with or without Cd^2+^.

### RNA extraction, cDNA synthesis and RT-PCR

Cells were plated and cultured for 24–48 h up to a confluency of 50% prior to treatments and/or transfection (as described above), harvested by scraping and subjected to isolation of total RNA and synthesis of cDNA as previously described (Betten et al.  2018). Cell numbers used for seeding and multiwell culture plate formats are summarized in Suppl. Table 1 for all cell lines and assays. RNA was isolated from mouse cortex and medulla tissue samples using High Pure RNA Tissue Kit (Roche; cat. # 12033674001) according to manufacturer’s protocol prior to cDNA synthesis. PCR reactions were performed using specific primers and cycling protocols (Table 1). Primers were designed using PrimerBLAST software (NCBI) and/or taken from the literature and synthesized by Eurofins Genomics. Gel documentation and densitometry analysis were performed using Image Lab Software version 5.2 (Bio-Rad Laboratories), with correction for loading by the housekeeping gene glyceraldehyde-3-phosphate dehydrogenase (*Gapdh*).

**Table 1:** Protocols for reverse transcriptase-PCR

	*Gapdh*	*Hamp1*	*Cat*	*Cat*
Species	Mus musculus	Mus musculus	Mus musculus	Rattus norvegicus
Accession number	NM_001289726.1	NM_032541.2	NM_009804.3	NM_012520.2
Forward primer (5′–3′)	AGGGCTCATGACCACAGT	TTGCGATACCAATGCAGAAG	GCAGATACCTGTGAACTGTC	GCGAATGGAGAGGCAGTGTAC
Reverse primer (5′–3′)	TGCAGGGATGATGTTCTG	GGATGTGGCTCTAGGCTATGTT	GTAGAATGTCCGCACCTGAG	GAGTGACGTTGTCTTCATTAGCACTG
Reference	NCBI Primer-BLAST	van Swelm et al. (2016)	El Mouatassim et al. (1999)	Limaye et al. (2003)
Activation	5 min 95°C	5 min 95°C	5 min 95°C	5 min 95°C
Cycle number	18–22	30–34	35	26
Denaturation	30 s 94°C	30 s 94°C	45 s 94°C	30 s 94°C
Annealing	30 s 60°C	30 s 60°C	60 s 56°C	30 s 58°C
Extension	30 s 72°C	30 s 72°C	60 s 72°C	30 s 72°C
Final Extension	7 min 72°C	7 min 72°C	7 min 72°C	7 min 72°C
PCR product (bp)	112	125	229	652

### qPCR

Cells were grown to confluence, treated ± transfection (see above and Suppl. Table 1), harvested by scraping and subjected to RNA isolation and cDNA synthesis as previously described (Betten et al. 2018). Target sequences were amplified by qPCR, essentially as described (Betten et al. 2018), in a StepOnePlus Real-Time PCR System (Applied Biosystems), using KAPA SYBR FAST qPCR Master Mix Universal with high ROX reference dye (Sigma-Aldrich). The following primers were used for amplification: rat and mouse *Hamp1,* forward *5′-GCTGCCTGTCTCCTGCTT-3′,* reverse *5′-TTACAGCATTTACAGCAGAAGAGG-3′* (Kanamori et al. 2014); mouse *Hamp1*, forward *5′-TTGCGATACCAATGCAGAAG-3′,* reverse *5′-GGATGTGGCTCTAGGCTATGTT-3′* (van Swelm et al. 2016)*;* rat and mouse *Gapdh, forward 5′-AGGGCTCATGACCACAGT-3′,* reverse *5′-TGCAGGGATGATGTTCTG-3′* (NCBI Primer-BLAST); mouse *Gapdh*, forward *5′-CGGCCGCATCTTCTTGTG-3′,* reverse *5′-CCGACCTTCACCATTTTGTCTAC-3′* (NCBI Primer-BLAST*)*; mouse *Actb,* forward *5′-CGTGCGTGACATCAAAGAGAA-3′*, reverse *5′-GGCCATCTCCTGCTCGAA-3′* (Betten et al. 2018); mouse *Cat*, forward *5′-GCAGATACCTGTGAACTGTC-3′*, reverse *5′-GTAGAATGTCCGCACCTGAG-3′* (El Mouatassim et al. 1999); rat *Cat*, forward *5′-GCGAATGGAGAGGCAGTGTAC-3′*, reverse *5′-GAGTGACGTTGTCTTCATTAGCACTG-3′* (Limaye et al. 2003). Primers were used at 300 nM final concentration. The cycling conditions were activation at 95°C for 5 min followed by 40 cycles of 95°C for 3 s and 60°C for 30 s (56°C for mouse *Cat* and 58°C for rat *Cat*), with melt curve analysis to check amplification specificity. Gene expression levels were calculated according to the 2^−∆Cq^ method relative to the sample with the highest expression (minimum Cq) (Bustin et al. 2009). The data obtained were corrected to the expression of two stable reference genes: *Gapdh* and *Actb*.

### Surface biotinylation

Cell surface proteins were isolated using the Pierce™ Cell Surface Protein Isolation Kit (Thermo Fisher Scientific; cat. # 89881), with some modifications. After biotinylation and quenching, cells were disrupted by nitrogen pressure cavitation at 350 lb per square inch for 2.5 min in a cell disruptor (Parr Instrument Company) followed by centrifugation of the homogenate at 1000×*g* for 10 min to remove nuclei and cellular debris, and the resulting supernatant was centrifuged at 15,000×*g* for 15 min to remove mitochondria. The remaining membranes in the supernatant were pelleted at 100,000×*g* for 45 min, subjected to a 15 min wash with 250 mM KBr to remove membrane-associated proteins and enrich transmembrane proteins, and centrifuged again at 100,000×*g* for 45 min. The resulting pellet was resuspended in kit lysis buffer and isolation of biotinylated proteins was continued according to the manufacturer’s protocol. All procedures were carried out at 4°C.

### Immunoblotting

Sodium dodecyl sulfate polyacrylamide gel electrophoresis (SDS-PAGE) and immunoblotting were essentially performed according to standard procedures using rapid semi-dry transfer (Bio-Rad Laboratories Trans-Blot Turbo). Homogenization of cells and mouse kidney cortex or medulla was performed by sonication (Branson 450 Digital Sonifier) in isosmotic sucrose buffer supplemented with protease inhibitor cocktail (Sigma-Aldrich). Homogenates were centrifuged for 5 min at 500×*g* to remove unbroken cells, and the supernatant was collected for determination of protein concentrations by the Bradford method, using bovine serum albumin (BSA) as standard (Bradford 1976). Samples were mixed with Laemmli buffer and heated for 5 min at 95°C [or for 15 min at 65°C for immunodetection of divalent metal transporter 1 (DMT1)] and subjected to SDS-PAGE and immunoblotted. Primary anti-catalase (cat. # ab209211 or cat. # ab16731; 1:2,000) and anti-β-actin (cat. # A5316; 1:20,000) antibodies were obtained from Abcam and Sigma-Aldrich, respectively. Anti-PARP (cat. # 9542; Cell Signaling Technology) was diluted 1:3000. Anti-calcium channel Ca_V_3.1 (α1G) (Sigma-Aldrich; cat. # C2240) was diluted 1:400. A rabbit polyclonal anti-rat DMT1 directed against the peptide sequence MVLDPEEKIPDDGASGDHGDS (Ferguson et al. 2001) generated by ImmunoGlobe GmbH (designated 1102#3) detects all four major DMT1 isoforms and was used at a concentration of 0.5 μg/ml. Horseradish peroxidase-conjugated secondary antibodies were purchased from Jackson ImmunoResearch Europe Ltd and used at a dilution of 1:10,000. Immunoblots were developed using Immobilon ECL substrate (Millipore; cat. # WBKLS0500) and visualized on blue X-ray films (Carestream). Densitometry analysis was performed using FIJI/ImageJ software (Schneider et al. 2012).

### Catalase assay

Cells were plated and cultured for 24 h up to a confluency of 50% prior to treatments and/or transfection, as described above (see Suppl. Table 1). Cells were harvested by scraping into PBS and collected by centrifugation at 2000×*g* for 1.5 min at 4°C. Catalase activity was determined using a Catalase Fluorometric Detection Kit (Enzo Life Sciences, Inc.; cat. # ADI-907-027) according to the manufacturer’s instructions. Cell pellets were resuspended in 55 µl of 1X reaction buffer and lysed by five freeze/thaw cycles. To fall within the standard curve range, samples were diluted to 0.25–1.0 µg protein. Samples were measured at *λ*_ex_ 530 nm *λ*_em_ 590 nm in a Mithras LB 940 Multimode Microplate Reader (Berthold Technologies) by repeated measurements every minute for 10 min. Catalase activity was determined from the standard curve performed in each experiment.

### Cell viability and cell death assays

Cells were plated and cultured for 24–72 h prior to treatments and/or transfection, as described above (see Suppl. Table 1). Cell viability was determined by the MTT test, as previously described (Lee et al. 2005). Trypan blue exclusion was used as a test for necrosis. Detached cells in the medium and adherent cells were collected, diluted 1:2 with 0.4% trypan blue and automatically counted (Countess II FL, Thermo Fisher Scientific). Apoptosis was detected using the APOSTRAND™ ELISA Apoptosis Detection Kit (Enzo Life Sciences, Inc.; cat. # BML-AK120) according to the manufacturer’s protocol. The APOSTRAND™ ELISA is based on the sensitivity of DNA in apoptotic cells to formamide denaturation and the detection of the denatured DNA with a monoclonal antibody to single-stranded DNA (ssDNA). Treatments were performed in FBS-free medium without phenol red. FBS-free medium without phenol red or PBS were used as negative controls whereas ssDNA included in the APOSTRAND™ ELISA served as a positive control.

### Immunofluorescence microscopy

0.5–6 × 10^4^ mIMCD_3_ or mCCD(cl.1) cells/cm^2^ were plated onto untreated or polylysine-treated glass coverslips in standard growth medium and stained 1–3 days later, essentially as described previously (Wolff et al. 2008). Unless otherwise indicated, all immunostaining procedures were performed at RT. In brief, cells were washed 2–3 times with Ca^2+^ and Mg^2+^ containing PBS, fixed with 2–4% paraformaldehyde (PFA) in PBS for 10–15 min, permeabilized with 0.1–0.5% Triton X-100 for 15 min (or 1% SDS for 5 min for hepcidin) and blocked with 1% bovine serum albumin in PBS (BSA-PBS) for 30 min. Cells were incubated with rabbit polyclonal anti-Ca_V_3.1 (Sigma-Aldrich; cat. # C2240; 1:250) and diluted in BSA-PBS overnight at 4°C. Alternatively, coverslips were stained either for metallothionein (MT) with mouse anti-MT clone E9 antibody (Dako; cat. # M0639; 1:500) in BSA-PBS for 90 min, or with rabbit polyclonal anti-hepcidin antibody (Abcam; cat. #ab30760; 1:100) in BSA-PBS for 2 h. After washout of primary antibodies, coverslips were incubated with anti-mouse or anti-rabbit Alexa Fluor® 488-conjugated secondary antibody (Thermo Fisher Scientific; cat. # A-11029 or A-11008; 1:400–1:500) in BSA-PBS for 45–60 min at RT. Nuclei were counterstained with 0.8 μg/ml 2′-(4-ethoxyphenyl)-5-(4-methyl-l-piperazinyl)-2,5′-bi-1H-benzimidazole, 3HCl (H-33342) (Calbiochem) for 5 min, and coverslips mounted with DAKO fluorescence mounting medium (Dako).

Conventional fluorescence microscopy was performed using a Zeiss Axiovert 200M epifluorescence microscope (Carl Zeiss) equipped with Fluar 40x/N.A. 1.3 and 100x/N.A. 1.3 oil immersion objectives, a Sola SM II light engine (Lumencor), filters for Alexa Fluor^®^ 488 (green) and H-33342 (blue) with excitation/emission wavelengths of 480 ± 20/535 ± 25 nm, and 360 ± 20/460 ± 25 nm, respectively, and a Cool-SNAP ES CCD camera (Roper Scientific) or a pco.panda 4.2 camera (PCO). Images were acquired at fixed exposure times at the focal plane of highest contrast and analyzed with MetaMorph software (Universal Imaging), as described elsewhere (Abouhamed et al.  2006), unless otherwise indicated. Confocal images were acquired using a TCS SP5 laser scanning confocal microscope (Leica) with a Plan-Apochromat 63x/N.A.1.4 oil immersion objective (Leica) and an argon (488 nm) laser and analyzed using LAS AF software.

### Immunofluorescence staining of tissue sections

For immunostaining, kidneys were cut into pieces and fixed with PLP (periodate/lysine/paraformaldehyde) overnight. Subsequently, tissue was cryoprotected in 15% and 30% sucrose, frozen in liquid nitrogen, and cut into 10 µm cryosections. Cryosections were washed with PBS, treated with 1% SDS/PBS for 5 min, blocked with 1% BSA-PBS for 15 min at RT and incubated with primary anti-catalase rabbit antibody (Abcam; cat. # ab209211; 1:1,000) in blocking solution overnight at 4°C. After washing with PBS, slides were incubated with donkey anti-rabbit IgG Alexa Fluor 594 (Thermo Fisher Scientific; cat. # A-11012; 1:400) for 1 h at RT. Slides were washed with PBS and mounted with Fluoromount-G (Southern Biotech; cat. # 0100-20) for nuclear staining. Slides were viewed using a TCS SP8 confocal laser scanning microscope with Plan-Apochromat CS2 20x/0.75NA IMM or CS2 40x/1.30NA oil immersion objective (Leica) and an OPSL (552 nm) laser.

### Detection of ROS formation using CellROX™ green

mIMCD_3_ cells were plated on glass coverslips and cultured for 72 h up to a confluency of 80% prior to treatments and/or transfection, as described above (see also Suppl. Table 1). Cells were incubated with 5 µM CellROX™ Green reagent (Thermo Fisher Scientific; cat. # C10444) for 30 min at 37°C. After washing with PBS containing Ca^2+^/Mg^2+^, cells were fixed with 3.7% PFA for 15 min at RT, washed with PBS, nuclei were counterstained with 0.8 μg/ml H-33342 for 5 min, and coverslips were mounted with DAKO fluorescence mounting medium. Images were acquired with fixed exposure times at the focal plane of highest contrast and were analyzed with MetaMorph, as described above.

### Quantification of fluorescence images

Quantification of fluorescence images was carried out using FIJI/Image J (Schindelin et al.  2012) or MetaMorph software, as described previously (Wolff et al. 2006). In brief, intensity thresholds were set to exclude cell-free regions. The mean fluorescence intensity per image was divided by the number of nuclei in the image to obtain average cellular fluorescence intensity. To correct for endogenous auto-fluorescence, intensity of negative control images, i.e., cells stained without primary antibody, was subtracted from all mean values.

### Statistics

Unless otherwise indicated, the experiments were repeated at least three times with independent cultures. Statistical analyses were performed with GraphPad Prism v. 5.01 software (GraphPad Software Inc.) and all data sets were tested for parametric/non-parametric distribution. Bar diagrams showing means ± SD were used for parametric data sets and box plots presenting 25 and 75 percentiles and median (horizontal line) for non-parametric data sets, unless otherwise indicated. Statistical comparison between two groups was performed using Student’s unpaired t-test or Mann–Whitney test, if groups were parametric or non-parametrically distributed, respectively. If more than two parametric groups were compared, one-way ANOVA with Bonferroni post hoc test was applied, while Kruskal–Wallis test with Dunn’s post hoc test was used for non-parametric groups. Results with *p* < 0.05 were considered statistically significant.

## Results

To investigate the role of hepcidin in Fe^2+^- and Cd^2+^-induced oxidative stress and damage at the cellular level, we first tested different rodent cell lines derived from several nephron segments. In rat PT [WKPT-0293 Cl.2; (Woost et al. 1996)], mouse cortical collecting duct (CCD) [mCCD(cl.1); (Gaeggeler et al. 2005)], and mouse inner medullary collecting duct (IMCD) [mIMCD_3_; (Rauchman et al. 1993)] cell lines, *Hamp1* gene expression was measured using primer pairs common to mouse and rat *Hamp1 *(see qPCR Methods). The highest mRNA expression levels were detected in mIMCD_3_ cells (Suppl. Fig. 1), which aligns with hepcidin protein levels in different segments of the mammalian nephron (Kulaksiz et al. 2005).

**Fig. 1:**
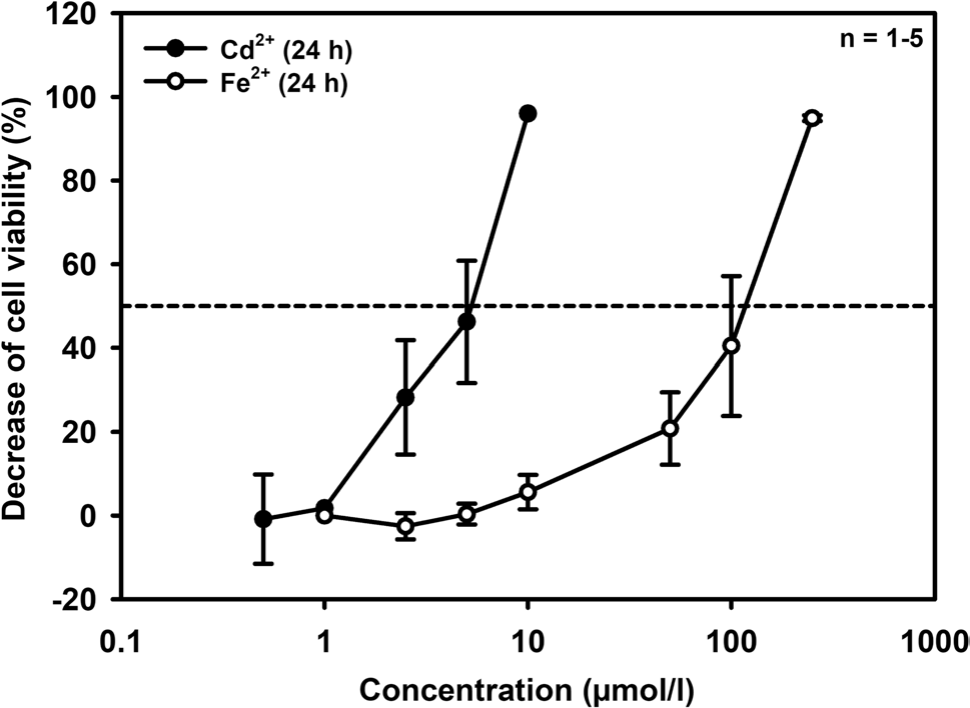
Viability of mouse inner medullary collecting duct cells (mIMCD_3_) exposed to different Cd^2+^ and Fe^2+^ concentrations for 24 h. Cell viability was determined by MTT assay. Means ± SD are shown with the exception of the highest concentration used

**Fig. 2:**
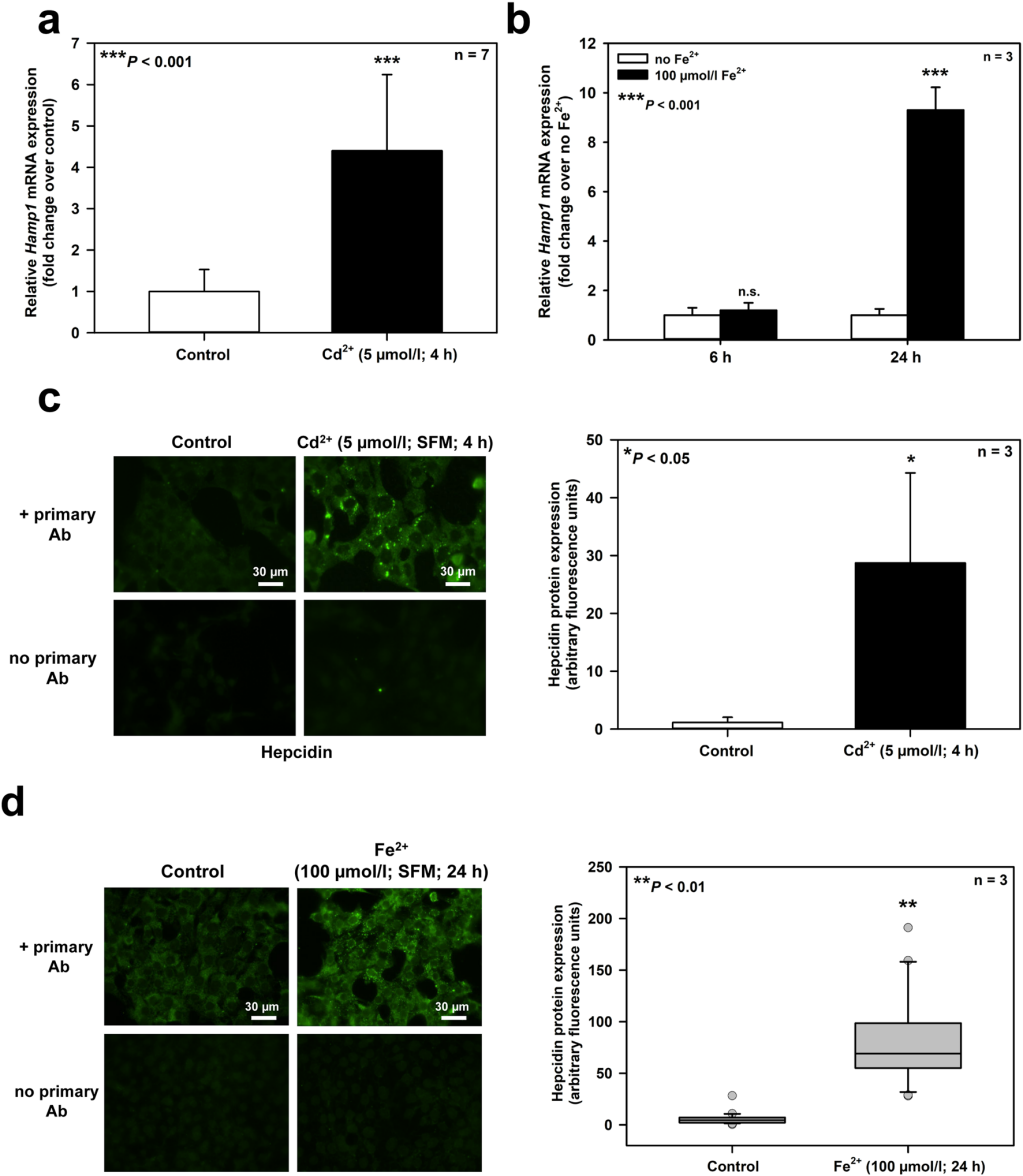
Cd^2+^ and Fe^2+^ increase hepcidin expression in mIMCD_3_ cells. Quantitative real-time PCR of *Hamp1* transcript levels in cells exposed to Cd^2+^ (**a**) or Fe^2+^ (**b**) for different times. Means ± SD are plotted. n.s. = not significant. Representative immunofluorescence microscopy images of hepcidin protein expression in cells exposed to Cd^2+^ (**c**, left) or Fe^2+^ (**d**, left) for different times. Immunofluorescence data were quantified in cells exposed to Cd^2+^ **(c**, right**)** or Fe^2+^ **(d**, right). Means ± SD are shown in **(c)** and box plots presenting 25 and 75 percentiles, median (horizontal line) and single experimental values in **(d)**. Statistical analyses plotted compare control versus experimental conditions using unpaired Student’s *t*-test **(a, b, c)** or non-parametric Mann–Whitney-test **(d)**

When mIMCD_3_ cells were exposed to Cd^2+^ or Fe^2+^ for 24 h, both metal ions decreased cell viability, although with different potencies. Cd^2+^ displayed an *EC*_50_ of about 5 μmol/l, whereas Fe^2+^ was less potent with an *EC*_50_ of about 100 μmol/l (Fig. 1) (equimolar concentrations of Fe^3+^ had no effect). Interestingly, Cd^2+^ and Fe^2+^ at *EC*_50_ concentrations increased hepcidin expression of mIMCD_3_ cells at both the mRNA and protein expression level, as assayed by immunofluorescence microscopy (Fig. 2), suggesting an adaptive response to metal ion stress and death. Cd^2+^ (5 μmol/l) appeared to be more effective than 100 μmol/l Fe^2+^ because Cd^2+^ rapidly (46 h) induced hepcidin expression (Figs. 2a, 2c). In contrast, Fe^2+^ had no effect at 6 h but after 24 h showed a similar magnitude of induction as Cd^2+^ (Figs. 2b, 2d). Of note, use of ascorbate in the Cd^2+^ experiments (ascorbate was used to prevent oxidation to Fe^3+^ in the Fe^2+^ experiments) did not interfere with *Hamp1* expression (123 ± 13% in 6 experiments, compared to respective *Hamp1* expression with Cd^2+^ but without ascorbate) (*data not shown*).

**Fig. 3:**
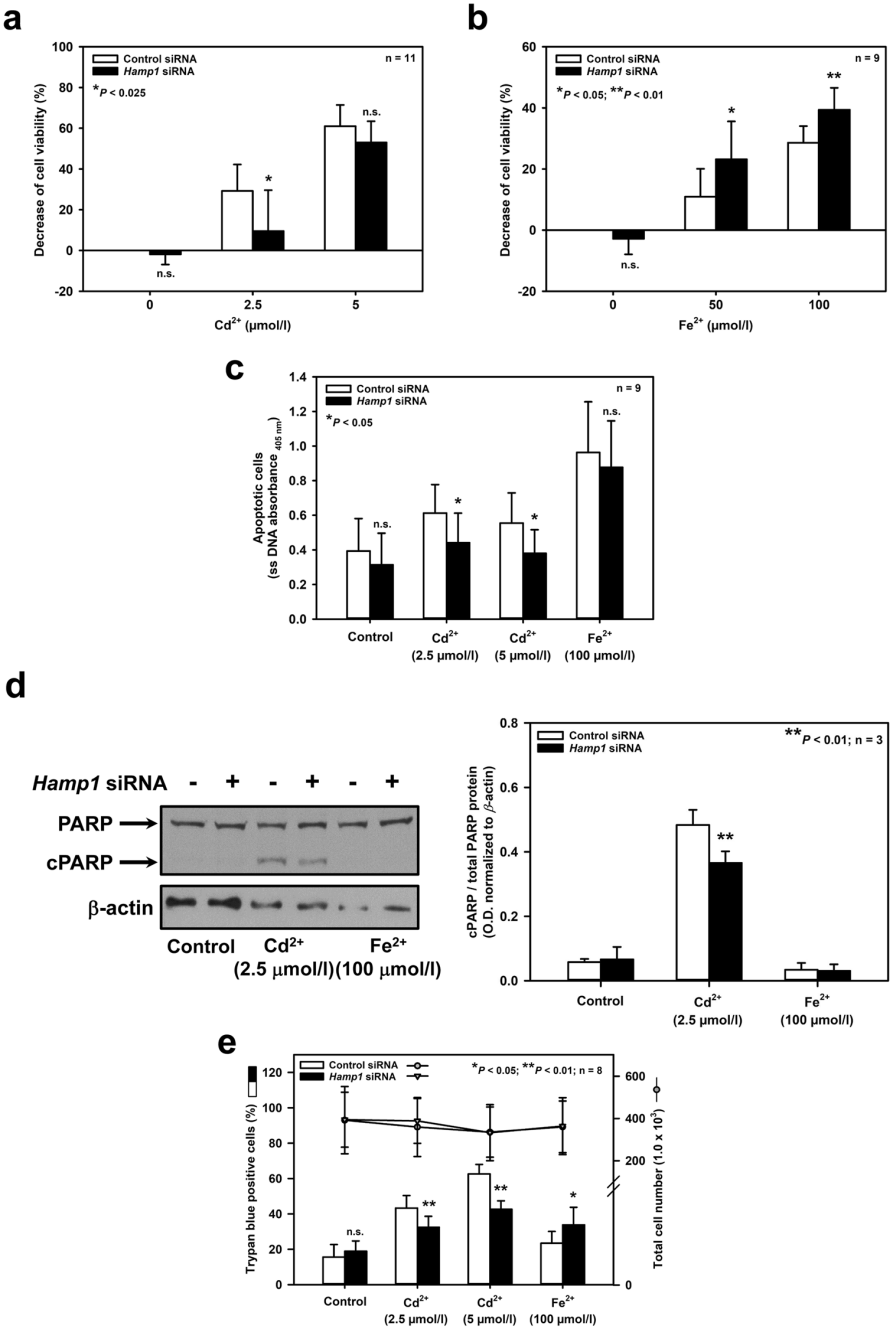

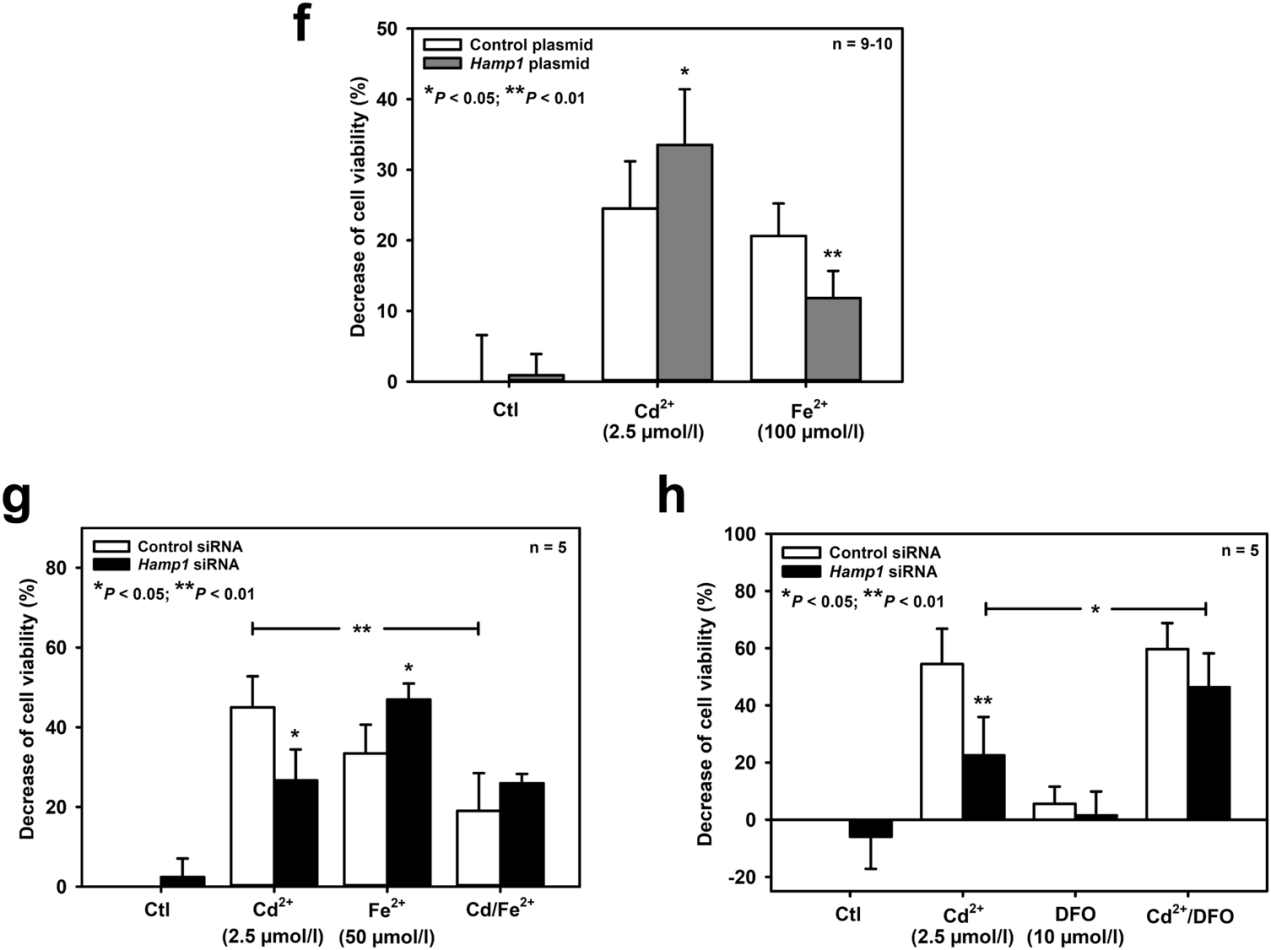
Opposing effects of hepcidin on cell fate of mIMCD_3_ cells exposed to Cd^2+^ or Fe^2+^ and protective role of iron on Cd^2+^-induced death. Hepcidin was either silenced by *Hamp1* siRNA **(a–e, g, h)**, or overexpressed by *Hamp1* plasmid transfection **(f**), as described in the *Methods*. Cell viability was determined by MTT assay **(a, b, f–h)**, apoptotic cell death by detection of single stranded (ss) DNA **(c)** or PARP cleavage (cPARP) **(d)**, and necrosis by measurement of trypan blue uptake as percentage of total cell number **(e)**. Cd^2+^, Fe^2+^ and/or the iron chelator desferrioxamine (DFO) were applied for 24 h prior to measurement of cell viability. Means ± SD are plotted. Statistical analyses compare either control siRNA/plasmid versus *Hamp1* siRNA/plasmid transfections using unpaired Student’s *t*-test **(a–c, e, f)**, or assess all experimental conditions using one-way ANOVA with Bonferroni post hoc test **(d, g, h)**. n.s. = not significant

To further clarify the role of hepcidin in cell injury induced by Cd^2+^ and Fe^2+^ in mIMCD_3_ cells, *Hamp1* was effectively silenced by RNAi, as shown by complete eradication of Cd^2+^-induced upregulation of *Hamp1* (Suppl. Figs 2a, 2b). Under these conditions, cell viability experiments were repeated in mIMCD_3_ cells. Surprisingly, *Hamp1* silencing had opposite effects on toxicity of both metal ions after 24 h exposure: Cd^2+^ toxicity was reduced (Fig. 3a) whereas Fe^2+^ toxicity was exacerbated (Fig. 3b). As an endpoint of cell viability/death, the MTT assay does not discriminate between apoptosis, necrosis, or decreased proliferation. Therefore, we complemented these observations with specific tests for apoptosis and necrosis, which essentially confirmed the MTT data: *Hamp1* silencing decreased both apoptosis (Figs. 3c, 3d) and necrosis (Fig. 3e) induced by Cd^2+^, whereas it increased necrosis in Fe^2+^-treated mIMCD_3_ cells (Fig. 3e). Transfection of a *Hamp1* plasmid in mIMCD_3_ cells for 24 h increased *Hamp1* expression by about twofold (Suppl. Fig. 2c) and had opposite effects on viability of mIMCD_3_ cells exposed to Cd^2+^ or Fe^2+^, when compared to *Hamp1* silencing (see Figs. 3a, 3b): Hepcidin overexpression further increased Cd^2+^-induced but reduced Fe^2+^-induced cell death (Fig. 3f).

**Fig. 4:**
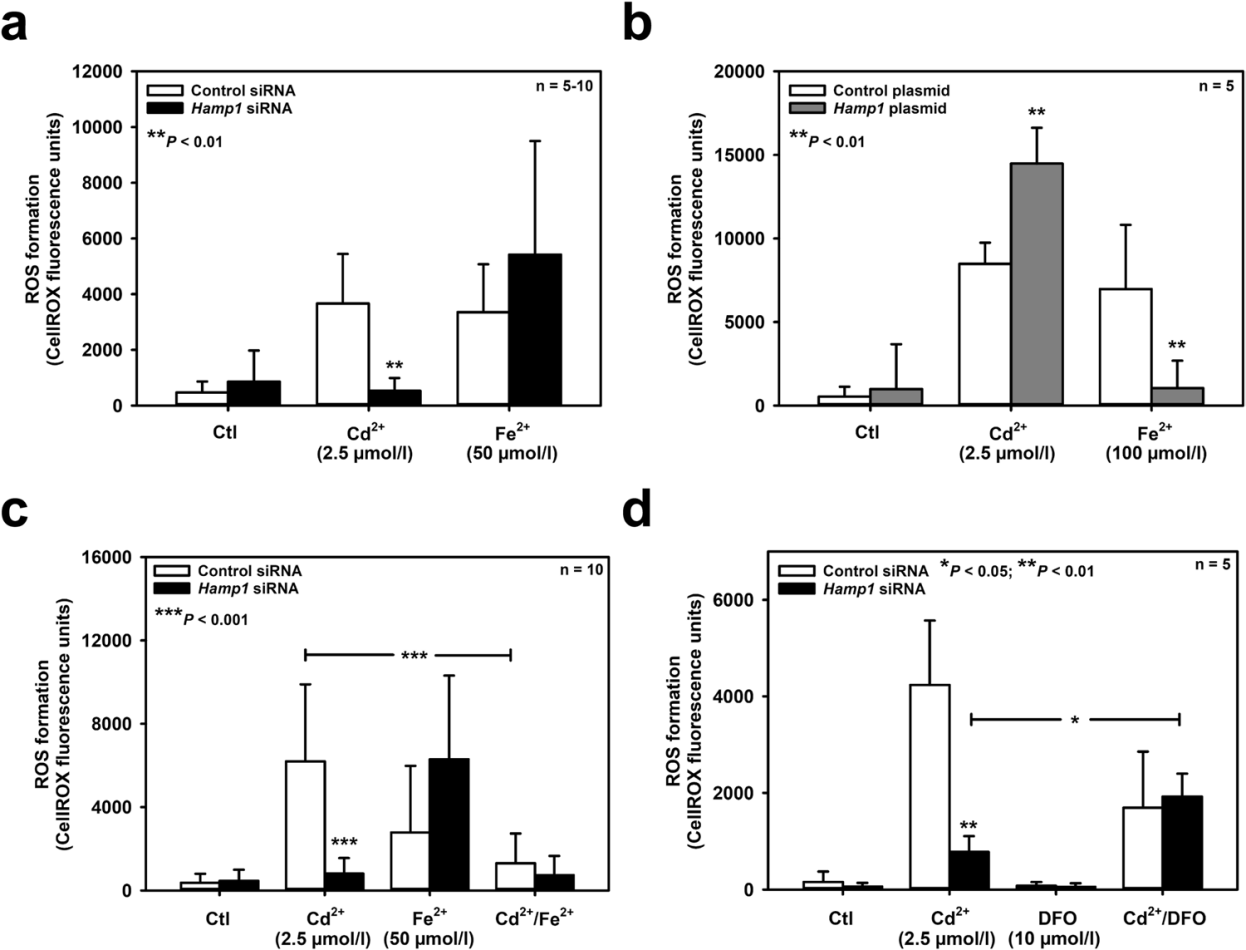
Diverging effects of hepcidin on ROS formation induced by Cd^2+^ and Fe^2+^ and protective role of iron on Cd^2+^-induced oxidative stress in mIMCD_3_ cells. Hepcidin was either silenced by *Hamp1* siRNA **(a, c, d)** or overexpressed by *Hamp1* plasmid transfection **(b)**, as described in the *Methods*. ROS formation was assayed with a fluorimetric kit. Cd^2+^, Fe^2+^ and/or the iron chelator desferrioxamine (DFO) were applied for 24 h prior to measurement of ROS formation. Means ± SD are plotted. Statistical analyses compare either control siRNA/plasmid versus *Hamp1* siRNA/plasmid transfections using unpaired Student’s *t*-test **(a, b)**, or evaluate all experimental conditions using one-way ANOVA with Bonferroni post-hoc test **(c, d)**

Hepcidin is protective against kidney cell injury associated with increased formation of ROS (Scindia et al. 2015; van Swelm et al. 2018). Furthermore, both Cd^2+^ and Fe^2+^ damage cells by inducing oxidative stress (reviewed in Cuypers et al. 2010; Dixon and Stockwell 2014). Hence, the role of hepcidin in oxidative stress elicited by Cd^2+^ and Fe^2+^ in mIMCD_3_ cells was investigated. As shown in Fig. 4a, both Cd^2+^ (2.5 μmol/l) and Fe^2+^ (50 μmol/l) increased ROS formation to a similar extent after 24 h exposure, as detected with CellROX™ Green. However, *Hamp1* silencing decreased ROS production induced by Cd^2+^ whereas it increased ROS with Fe^2+^ exposure. Overexpressing hepcidin in mIMCD_3_ cells augmented Cd^2+^- but abolished Fe^2+^-induced ROS formation (Fig. 4b). ROS measurements mirrored MTT cell viability data (Fig. 3), indicating that Cd^2+^- and Fe^2+^-induced oxidative stress mediates cell death in mIMCD_3_ cells. Yet hepcidin had opposite effects on Cd^2+^- and Fe^2+^-induced ROS formation and consequent cell death, namely protection against Fe^2+^ but aggravation of Cd^2+^-mediated oxidative stress and death.

Hepcidin can chelate endogenous metal ions, in particular Fe^2+^ (Abbas et al. 2018; Farnaud et al. 2006, 2008; Gerardi et al. 2005). If so, the expression level of cellular hepcidin should determine the levels of endogenous redox active iron. This would explain the protective effects of hepcidin overexpression (Figs. 3f, 4b), and increased damage and ROS formation by *Hamp1* silencing (Figs. 3b, 3e, 4a) in Fe^2+^-exposed cells. In contrast, opposite results obtained in Cd^2+^-exposed cells (Figs. 3a, 3c–f, 4a, 4b) make it unlikely that hepcidin also chelates Cd^2+^ in a similar manner to Fe^2+^. Cd^2+^ damages proteins and cells by competing with and displacing Fe^2+^ from various binding sites (reviewed in Moulis 2010). We therefore hypothesized that increased endogenous Fe^2+^ in *Hamp1*-silenced cells prevents its displacement by Cd^2+^ and cell injury (Figs. 3a, 3c–e). Conversely, decreased endogenous Fe^2+^ by hepcidin overexpression promotes Cd^2+^ displacement and cell damage (Fig. 3f).

To test this concept, the effect of exogenously added Fe^2+^ (50 μmol/l) was investigated on viability of control and *Hamp1*-silenced mIMCD_3_ cells exposed to Cd^2+^ (2.5 μmol/l, 24 h). Addition of Fe^2+^ increased cell viability of Cd^2+^ exposed cells transfected with control siRNA, but had no additional effect in *Hamp1*-silenced cells (Fig. 3g), suggesting a cellular mechanism protecting against Cd^2+^ toxicity that needs iron. We then performed the opposite experiment by depleting chelatable endogenous cellular iron with the iron chelator desferrioxamine (DFO) (Keberle 1964), which does not bind Cd^2+^ (Harrington et al. 2012). DFO (10 μmol/l) abolished the protective effect of *Hamp1* silencing on Cd^2+^ damage (Fig. 3h), indicating that increased endogenous chelatable iron protects against Cd^2+^ damage in *Hamp1*-deficient cells. ROS formation induced by Cd^2+^ was also measured under conditions of exposure to exogenously added Fe^2+^ or DFO: addition of Fe^2+^ (50 μmol/l) decreased Cd^2+^-induced ROS formation in control cells, with no further effect by *Hamp1* silencing (Fig. 4c). With DFO, *Hamp1*-silenced cells showed a significant increase of Cd^2+^-induced ROS formation, when compared to the respective controls without DFO (Fig. 4d).

ROS metabolizing enzymes require metal ions as co-factors for their activity. In contrast to superoxide dismutases and glutathione peroxidases, which are not iron-dependent (Halliwell and Gutteridge 2015), catalases are iron-dependent enzymes (Deisseroth and Dounce 1970) that convert hydrogen peroxide (H_2_O_2_) into oxygen and water (Ratliff et al. 2016). Strikingly, their activity is increased or reduced by high or low cellular iron, respectively (Deisseroth and Dounce 1970; Macdougall 1972; Schultze and Kuiken 1941; Srigiridhar and Nair 1998), and disrupted by Cd^2+^, which results in increased ROS formation with Cd^2+^ (reviewed in Cuypers et al. 2010). Deep sequencing of micro-dissected nephron segments from rodent kidney demonstrated catalase mRNA (*Cat*) is mainly detected in the S1-S3 segments of the PT, but significant levels of catalase can be detected in other nephron segments as well. In the CD, the highest expression was measured in the IMCD, although PT expression was about sixfold higher than in the IMCD (Lee et al. 2015). Expression of *Cat* mRNA by qPCR was ~ 2.5-fold higher in WKPT-0293 Cl.2 than in mIMCD_3_ cells (Suppl. Table 2), which was confirmed at the protein level by immunoblotting. The expression of catalase protein by immunofluorescence staining of mouse kidney sections (Suppl. Fig. 3a) as well as by immunoblotting of crudely separated mouse kidney cortex and medulla (Suppl. Fig. 3b) confirmed published differential expression measured in rodent cortical and medullar nephron segments at the mRNA (Lee et al. 2015) and protein levels (Muse et al. 1994). Moreover, catalase activity in rodent kidney cortex and medulla (Gonzalez-Flecha et al. 1993) correlated with mRNA expression levels [see above; (Lee et al. 2015)] and matched catalase activity in PT WKPT-0293 Cl.2 cells, which was approximately fourfold higher than in mIMCD_3_ cells (Suppl. Table 2).

**Fig. 5:**
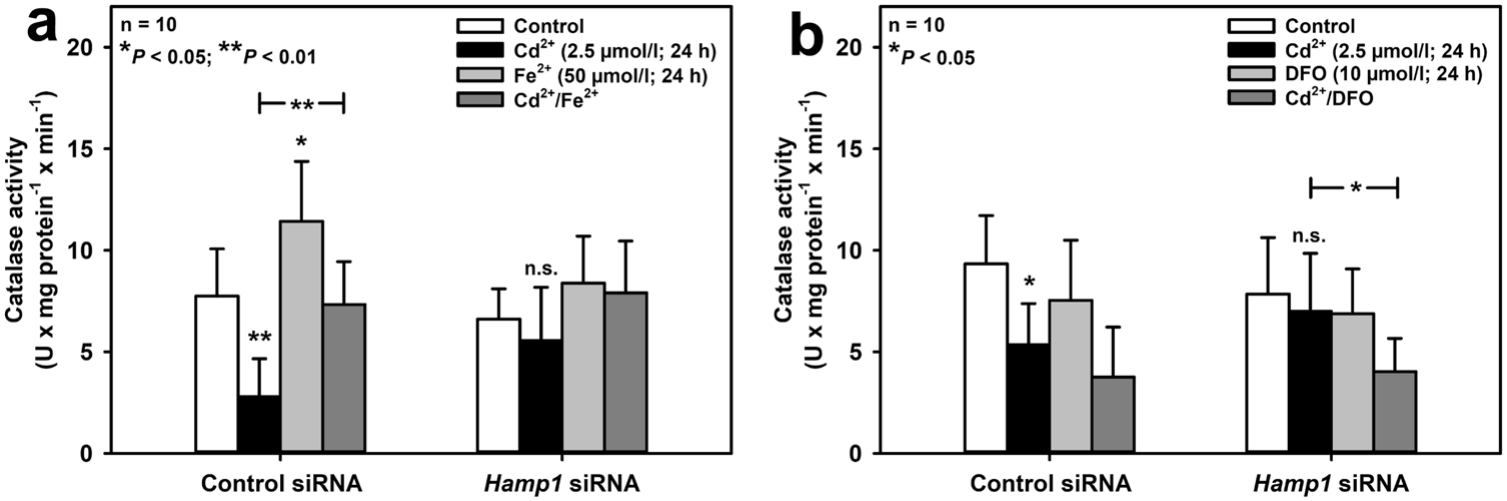
Opposite effects of hepcidin silencing on catalase activity of mIMCD_3_ cells exposed to Cd^2+^ or Fe^2+^ and protective role of iron on Cd^2+^-induced catalase dysfunction. Cells were incubated with divalent metal ions or DFO for 24 h prior to measuring catalase activity with a fluorometric assay. The effects of Fe^2+^ **(a)** or of the iron chelator desferrioxamine (DFO) **(b)** on catalase activity were determined in the absence or presence of Cd^2+^ in control or *Hamp1*-silenced cells. Means ± SD are shown. Statistical analysis evaluates all experimental conditions with each other using one-way ANOVA with Bonferroni post-hoc test. n.s. = not significant

The effect of Cd^2+^ on catalase function and the impact of exogenously added Fe^2+^ or DFO were investigated in mIMCD_3_ cells with or without *Hamp1* silencing. As shown in Suppl. Fig. 4, except for Fe^2+^ increasing *Cat* mRNA, none of the experimental manipulations performed in this study significantly affected *Cat* mRNA expression. Yet exposure of cells to Cd^2+^ for 24 h strongly decreased catalase activity, whereas Fe^2+^ enhanced catalase activity (Fig. 5a), which confirms recent observations in plants (Biyani et al. 2019). The latter may be partly accounted for by the induction of *Cat* gene expression by Fe^2+^ (see Suppl. Fig. 4 and Piloni et al. 2016). Co-exposure with Fe^2+^ prevented the reduction of catalase activity induced by Cd^2+^. And *Hamp1* silencing had the same protective effect as addition of Fe^2+^ (although neither *Hamp1* silencing nor overexpression affected *Cat* mRNA levels; see Suppl. Fig. 4) on Cd^2+^-induced inhibition of catalase activity (Fig. 5a). DFO prevented the rescue of catalase activity by *Hamp1* silencing in Cd^2+^-treated cells (Fig. 5b), which once more supports the concept that hepcidin binds iron (Farnaud et al. 2006, 2008; Gerardi et al. 2005).

**Fig. 6:**
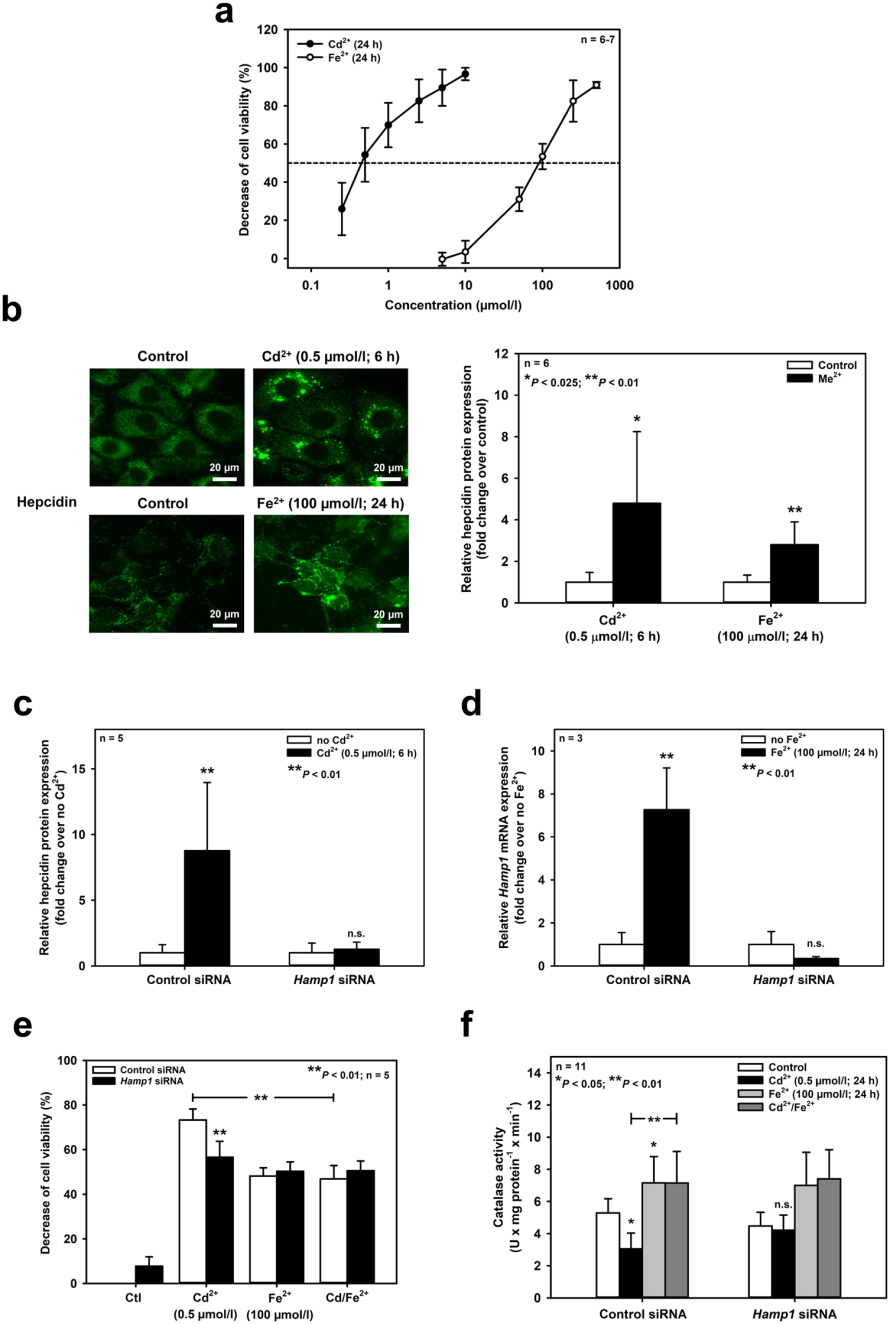
Cd^2+^ and Fe^2+^ increase hepcidin expression, which sensitizes mouse cortical collecting duct cells to Cd^2+^-induced death and catalase dysfunction whereas co-exposure with Fe^2+^ or *Hamp1* silencing protect against Cd^2+^-induced damage. (**a**) Viability of mCCD(cl.1) cells exposed to various Cd^2+^ and Fe^2+^ concentrations by MTT assay. (**b**) Representative immunofluorescence microscopy images of hepcidin protein in cells exposed to Cd^2+^ or Fe^2+^ (left) and quantification of immunofluorescence data (right). Statistical analysis compares control versus experimental conditions using unpaired Student’s *t*-test. (**c, d**) Efficient hepcidin silencing with *Hamp1* siRNA. Cd^2+^ upregulation of hepcidin protein by immunofluorescence microscopy **(c)**, or Fe^2+^-mediated induction of *Hamp1* mrNA by quantitative real-time PCR **(d)** were abolished by transient transfection of *Hamp1* siRNA. Satistical analyses compare controls versus metal ion-treated cells using unpaired Student’s *t*-test. (**e, f**) Effects of Fe^2+^ and/or hepcidin silencing with *Hamp1* siRNA on cell viability **(e)** or catalase activity **(f)** measured in the absence or presence of Cd^2+^. Viability was measured by MTT assay and catalase activity with a fluorimetric assay. Statistical analysis evaluates all experimental conditions with each other using one-way ANOVA with Bonferroni post-hoc test. n.s. = not significant

**Fig. 7:**
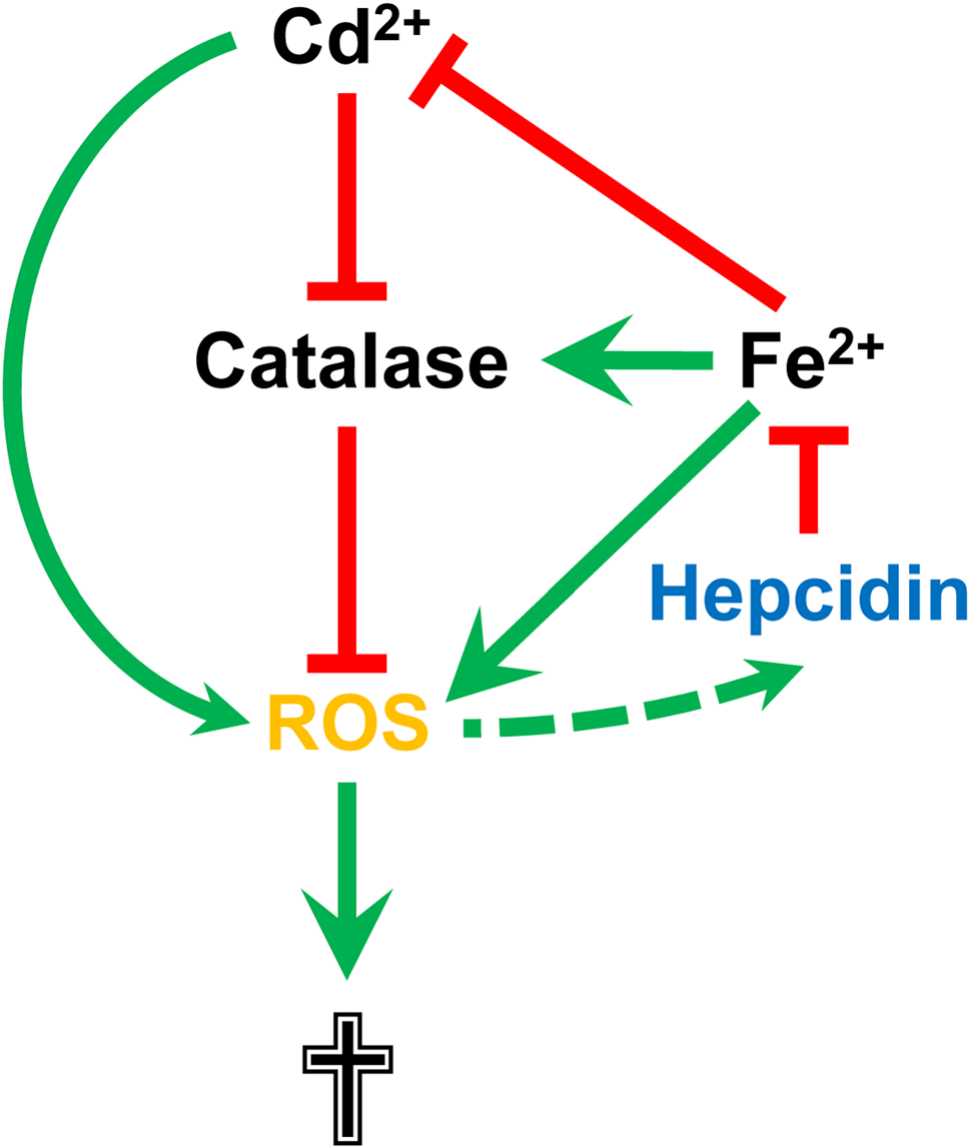
Model of the influence of hepcidin, Cd^2+^ and Fe^2+^ on ROS formation, catalase activity and cell death in renal collecting duct cells. Both Cd^2+^ and Fe^2+^ increase formation of reactive oxygen species (ROS) in renal collecting duct (CD) cells and iron-dependent catalase degrades ROS. ROS cause cell death but also induce upregulation of hepcidin, which chelates Fe^2+^, but not Cd^2+^. Cd^2+^ inactivates catalase by displacing iron from catalase. Fe^2+^ reduces Cd^2+^-induced catalase inactivation, while hepcidin upregulation protects CD cells against Fe^2+^-induced oxidative stress, but sensitizes them to Cd^2+^-induced catalase dysfunction, ROS formation and death. For further details, see main text. Green arrows indicate stimulatory, red arrows inhibitory effects. Solid lines represent experimental evidence from the study, dotted lines evidence from the literature

Hepcidin is also detected in the cortical collecting duct (Gaeggeler et al. 2005), though at a lower level (Kulaksiz et al. 2005) (see also Suppl. Fig. 1). To ascertain whether reduced levels of hepcidin also interfere with endogenous redox active ion levels, key experiments were executed in a model system, namely the mCCD(cl.1) cell line, derived from mouse cortical collecting duct (see Suppl. Fig. 1). As shown in Fig. 6a, Cd^2+^ or Fe^2+^ for 24 h decreased cell viability in a concentration-dependent manner. Cd^2+^ was about ten times more toxic in mCCD(cl.1) cells (*EC*_50_ ~ 0.5 μmol/l) than in mIMCD_3_ cells, whereas Fe^2+^ displayed similar toxicity in both cell lines (*EC*_50_ of about 100 μmol/l) (see Fig. 1). Cd^2+^ and Fe^2+^ at *EC*_50_ concentrations increased hepcidin mRNA and protein expression in mCCD(cl.1) cells, which was prevented by transient transfection with *Hamp1* siRNA (Fig. 6b–d and *data not shown*). Hepcidin silencing or co-exposure with Fe^2+^ reduced Cd^2+^, but not Fe^2+^ toxicity (Fig. 6e). These data further supported the hypothesis that hepcidin chelates protective Fe^2+^ in mCCD(cl.1) cells and that Fe^2+^ or *Hamp1* silencing rescue catalase dysfunction elicited by Cd^2+^, which was confirmed by the experiments shown in Fig. 6f. This further underlines the validity of the model shown in Fig. 7 for all nephron segments (and possibly other tissues) expressing hepcidin.

## Discussion

In the present study, we demonstrate distinct effects of hepcidin on Fe^2+^- and Cd^2+^-induced oxidative stress and death in mIMCD_3_ and mCCD(cl.1) cells (Fig. 7). Hepcidin protects against Fe^2+^-mediated oxidative cell injury but aggravates Cd^2+^-induced ROS formation and death (Figs. 3, 4).

Cd^2+^ is about 20–200 times more potent than Fe^2+^ to induce cell injury (Figs. 1, 3, 6a, 6e), ROS formation (Fig. 4), and hepcidin upregulation (Figs. 2, 6b–d) in mIMCD_3_ and mCCD(cl.1) cells. The different damaging potency of Cd^2+^ and Fe^2+^ could be explained by differences in the permeability of both metal ions in these cells as well as competition between Cd^2+^ and Fe^2+^ for uptake pathways in experiments where Cd^2+^ and Fe^2+^ were co-exposed. Two transport pathways that carry both Cd^2+^ and Fe^2+^ are expressed in rodent CD, namely DMT1 and the voltage-gated calcium channel Ca_V_3.1 (α_1_G) (Andreasen et al. 2000; Ferguson et al. 2001; Wareing et al.  2003) (reviewed in Thévenod and Wolff 2016). The localization of these proteins was investigated in mIMCD_3_ cells in which most experiments in this study were performed. DMT1 was not detected among mIMCD_3_ surface proteins, as determined by surface biotinylation and immunoblotting (Suppl. Fig. 5a), thus excluding DMT1 as a relevant uptake pathway for Cd^2+^ and Fe^2+^. In contrast, the calcium channel Ca_V_3.1 was expressed in mIMCD_3_ cells by immunofluorescence microscopy (Suppl. Fig. 5b, left) and mainly observed at the cell periphery, suggesting localization in the plasma membrane, which was confirmed by laser scanning confocal microscopy (Suppl. Fig. 5b, right) and surface biotinylation experiments (Suppl. Fig. 5c, arrow). Ca_V_3.1 shows a lower permeability for Fe^2+^ compared to Cd^2+^ (Lopin et al. 2012a, b), which could well explain the higher potency of Cd^2+^ compared to Fe^2+^ (Figs. 1, 6a), but cannot be the reason for Cd^2+^ and Fe^2+^ co-exposure almost abolishing comparable levels of Cd^2+^or Fe^2+^ toxicity and oxidative stress (Figs. 3g, 4c, 6e).

The protein MT is upregulated by Cd^2+^ in the kidney (Sabolic et al. 2010) and protects against Cd^2+^ toxicity by its strong Cd^2+^ chelating (Freisinger and Vasak 2013) and anti-oxidative properties (Ruttkay-Nedecky et al. 2013), but it lacks iron binding properties (Waalkes et al. 1984). To exclude the possibility that *Hamp1* silencing as well as increased or decreased endogenous iron might have altered the expression of MT, which would explain some of the observed Cd^2+^-induced changes of cell viability and ROS formation under those experimental conditions, immunofluorescence staining of endogenous MT was performed in mIMCD_3_ cells. Basal MT expression was low in mIMCD_3_ cells, while it increased by 5.5–8.3-fold (means of six experiments) upon exposure to 2.5 μmol/l Cd^2+^ for 24 h (Suppl. Fig. 6). In contrast, *Hamp1* silencing, Fe^2+^ (50 μmol/l) or DFO (10 μmol/l) did not have any effect on MT expression (Suppl. Fig. 6). Hence, MT expression does not contribute to the observed effects of these experimental manipulations.

Several protective roles have been attributed to local (renal) hepcidin in the context of metal ion toxicity. Hepcidin protects against cell death induced by hemoglobin (van Swelm et al. 2016, 2018), reduces oxidative stress in murine ischemia/reperfusion acute kidney injury (Scindia et al. 2015), or chelates endogenous metal ions, including Fe^2+^ and copper (Abbas et al. 2018; Farnaud et al. 2006, 2008; Gerardi et al. 2005). All three mechanisms are consistent with the Fe^2+^, but not Cd^2+^, effects described in this study (see Figs. 3–6). However, both Fe^2+^ and Cd^2+^ damage mIMCD_3_ cells by oxidative stress (Figs. 3, 4). To reconcile these seemingly disparate results, we hypothesized that endogenous iron levels are crucial for catalase function and Cd^2+^ damages catalase by displacement of iron (Moulis 2010). As heme-containing enzymes, catalases require iron for their activity (Deisseroth and Dounce 1970), and iron deficiency decreases their activity in various tissues, including the kidney (Macdougall 1972; Schultze and Kuiken 1941; Srigiridhar and Nair 1998). Hepcidin upregulation increases binding of cellular iron (Farnaud et al. 2006, 2008; Gerardi et al. 2005), thus reducing the redox active labile iron pool (LIP) (Kakhlon and Cabantchik 2002) and cell stress induced by Fe^2+^ overload (Figs. 3f, 4b), but enhances Cd^2+^ toxicity by chelating endogenous iron that is required for the function of catalase (see Figs. 5, 6f).

Obviously, hepcidin appears to have no singular role in the cell (see above). Moreover, scavenging of ROS seems unlikely as a protective mechanism of hepcidin because ROS formation is increased by Cd^2+^ in mIMCD_3_ cells despite hepcidin upregulation (Fig. 2) and Cd^2+^-induced ROS formation is decreased by *Hamp1* silencing (Fig. 4). Further, both systemic production of hepcidin as a liver hormone as well as tissue-specific expression as a local regulatory factor need to be considered to understand the manifold functions of hepcidin. In the kidney, hepcidin is not expressed in the PT but exclusively found in the distal nephron (Kulaksiz et al. 2005). There it appears to protect against iron overload by controlling luminal iron uptake via DMT1 downregulation, possibly involving an autocrine mechanism (Moulouel et al. 2013). Here we demonstrate an additional protective effect of intracellular hepcidin against iron overload in distal nephron cells. Hepcidin reduces ROS formation (Fig. 4) and cell death (Figs. 3, 6), likely through its role as an iron scavenger (Farnaud et al. 2006, 2008; Gerardi et al. 2005) thus decreasing cellular levels of the redox active LIP (Kakhlon and Cabantchik 2002). Hepcidin upregulation by Fe^2+^ (Figs. 2, 6b, 6d) could be an additional mechanism of protection of the distal nephron against iron damage; H_2_O_2_ is known to upregulate hepcidin in hepatocytes (Millonig et al.  2012), which could be the underlying mechanism accounting for hepcidin induction by Cd^2+^ and Fe^2+^. Systemic iron overload pathology mainly targets the PT in experimental animals (Richter 1980; Zainal et al. 1999) and humans (Landing et al. 1989; Pardo-Mindan et al. 1990) because the PT represents the preferential site of iron uptake, as it possesses the perfect machinery required for transepithelial iron reabsorption in the kidney (reviewed in Thévenod and Wolff 2016). Hence, lack of hepcidin expression in the PT (Kulaksiz et al. 2005) may be detrimental under conditions of increased PT iron reabsorption whereas catalase upregulation induced by iron (see Suppl. Fig. 4) would be protective, at least transiently.

Chronic Cd^2+^ exposure leads to accumulation of Cd^2+^ in various organs, in particular the kidney PT, because of the presence of major luminal uptake pathways for Cd^2+^ along with limited routes of basolateral exit and subsequent damage (reviewed in Prozialeck and Edwards 2012; Thévenod and Wolff 2016). MT is the major protective mechanism against Cd^2+^ toxicity in the PT and other tissues (reviewed in Klaassen et al. 2009). In addition, catalase is highly expressed in the PT (Lee et al. 2015; Muse et al. 1994), mainly in peroxisomes, but also in the cytosol (Kalmbach and Fahimi 1978; Litwin et al. 1988). Hence, disruption of PT catalase activity by both low iron and/or increased Cd^2+^ will favor dysfunction of the PT, but also the distal nephron, as shown in this study for mIMCD_3_ cells and mCCD(cl.1) cells. Interestingly, catalase activity in the three cell lines originating from different nephron segments [WKPT-0293 Cl.2 > mIMCD_3_ > mCCD(cl.1)] (see Suppl. Table 1) appears to inversely correlate with Cd^2+^ toxicity [WKPT-0293 Cl.2 < mIMCD_3_ < mCCD(cl.1)] (see Figs. 1, 6a and Nair et al. 2015). This is not the case for Fe^2+^ toxicity (see Figs. 1, 6a), suggesting that catalase activity is an important contributor to protection against Cd^2+^-induced damage of the kidney.

The absence of hepcidin in the PT could further protect against Cd^2+^ toxicity, as shown in *Hamp1*-silenced mIMCD_3_ cells and mCCD(cl.1) cells (Figs. 3a, 3c–e, 6e). Cd^2+^ also accumulates in the distal nephron (Nagamine et al. 2007; Torra et al. 1994; Wang et al. 2009; Yoshida et al. 1998). The lower catalase (Lee et al. 2015; Muse et al. 1994) and higher hepcidin expression levels (Kulaksiz et al. 2005) (which may be further upregulated by Cd^2+^; see Figs. 2a–c, 6b, 6c) enhance Cd^2+^-induced ROS formation and hence Cd^2+^ toxicity (see Figs. 3f, 4b). Why then is Cd^2+^ nephrotoxicity less pronounced in the medullary distal nephron (Johri et al. 2010)? Cellular Cd^2+^ entry pathways [i.e., entry pathways for essential metal ions, such as Fe^2+^, Zn^2+^, Mn^2+^, Ca^2+^ and their complexes with proteins or peptides; reviewed in (Thévenod et al. 2019)] are comparable between kidney cortex and medulla. The relative resistance of the distal nephron to Cd^2+^ toxicity may result from its lower sensitivity to oxidative stress (Campos et al. 1993; Gonzalez-Flecha et al. 1993). Thus increased adaptive responses by oxidative and other stress-induced processes must underlie less prominent Cd^2+^ toxicity in the distal nephron (Johri et al. 2010), Consequently, the distal nephron displays an increased potential for adaptive responses by oxidative stress-induced factors [e.g., hypoxia-inducible factor-1α (HIF-1α), neutrophil gelatinase-associated lipocalin (NGAL), to name a few (van Swelm et al. 2018; Zou and Cowley 2003)]. Furthermore, the metabolic profile in the medullary segments shows largely anaerobic glycolysis due to a low partial pressure for O_2_ (McDonough and Thompson 2012) along with lower abundance of mitochondria. This particular redox status could protect the kidney medulla from Cd^2+^-induced oxidative stress resulting from mitochondrial damage (Thévenod et al. 2020; Lee et al. 2020), although antioxidant enzyme activities are concomitantly decreased in the medulla compared to the cortex (Thiab et al. 2015).

If hepcidin plays a role in determining cellular iron levels, as supported by the current study, it is imaginable that physiologically hepcidin expressed in the distal nephron could regulate the activity of iron-dependent enzymes and functions, e.g., cellular metabolism, ATP production, ROS formation and/or signaling. Interestingly, the distribution of peroxisomal enzymes, including catalase, along the nephron correlates with that of mitochondria, a major source of cellular ROS formation, as well as of Na^+^/K^+^-ATPase activity (Guder and Ross 1984). Peroxisomes and mitochondria relate functionally, as evidenced by their communication, cooperation and division machinery (Vasko 2016). Thus, hepcidin in the distal nephron could aid to adjust the metabolic and transport demands of the distal nephron under conditions of low tissue O_2_ partial pressure, as encountered in the kidney medulla. This requires verification in future studies.

In summary, as studied in renal mIMCD_3_ and mCCD(cl.1) cells, hepcidin binds Fe^2+^ but not Cd^2+^. Because Fe^2+^ and Cd^2+^ compete for functional binding sites in proteins, hepcidin affects their respective free metal ion pools and impacts differently on downstream processes, such as ROS formation, catalase activity and cell fate (see Fig. 7).

## Supplementary Information

Below is the link to the electronic supplementary material.

Supplementary file1 (PDF 2201 kb)

## Data Availability

The datasets used and/or analyzed during the current study are available from the corresponding authors on reasonable request.
