# Supplementary material for: Role of hepcidin in oxidative stress and cell death of cultured mouse renal collecting duct cells: protection against iron and sensitization to cadmium
Source: Arch Toxicol. 2021 Jun 28;95(8):2719–35. doi: 10.1007/s00204-021-03106-z (PMC8298330; doi:10.1007/s00204-021-03106-z)
Supplement: Supplementary file 1 — Supplementary file1 (PDF 2201 kb) [file 204_2021_3106_MOESM1_ESM.pdf]

Archives of Toxicology

Supplementary Figures 1-6 + Supplementary Table 1

Role of hepcidin in oxidative stress and cell death of renal medullary collecting duct cells: Protection against iron but sensitization to cadmium

S. Probst, J. Fels, N.A. Wolff, E. Roussa, R.P.L. van Swelm, W.-K. Lee, F. Thévenod

Institute of Physiology, Pathophysiology & Toxicology and ZBAF (Centre for Biomedical Education and Research), Faculty of Health, School of Medicine, Witten/Herdecke University, Stockumer Str 12 (Thyssenhaus), D-58453 Witten, Germany
E-mail: frank.thevenod@uni-wh.de

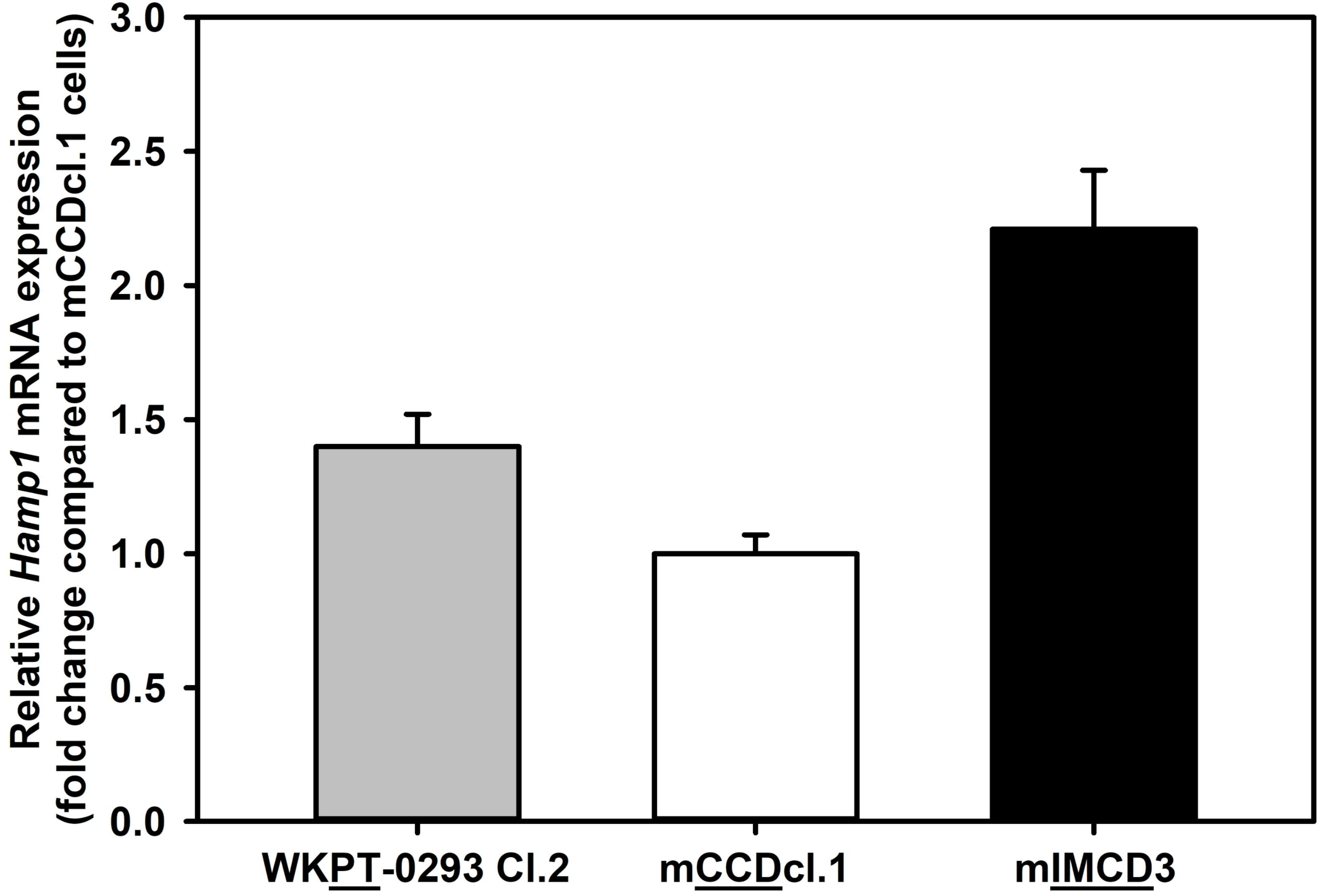

Suppl. Figure 1

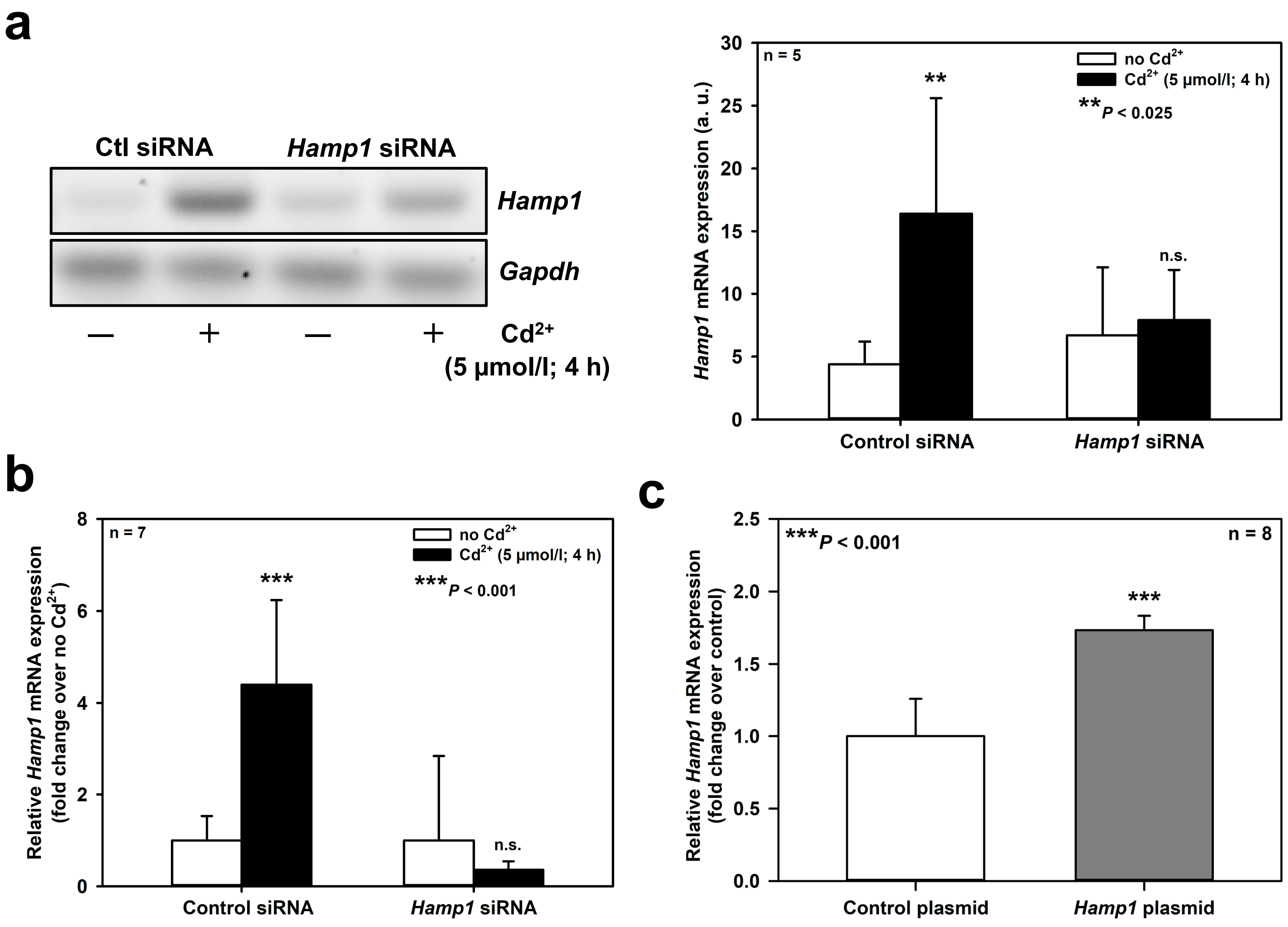

Suppl. Figure 2

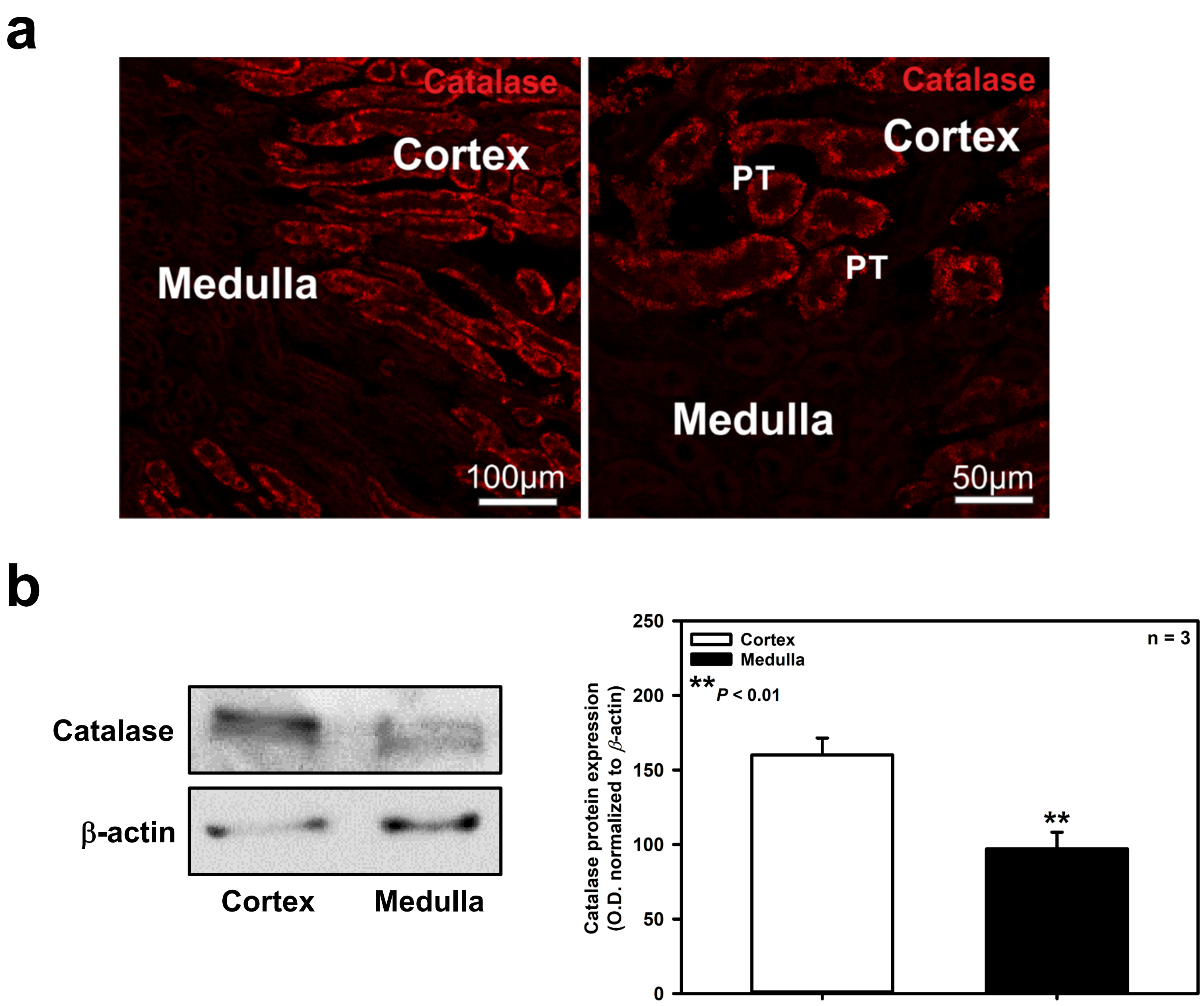

Suppl. Figure 3

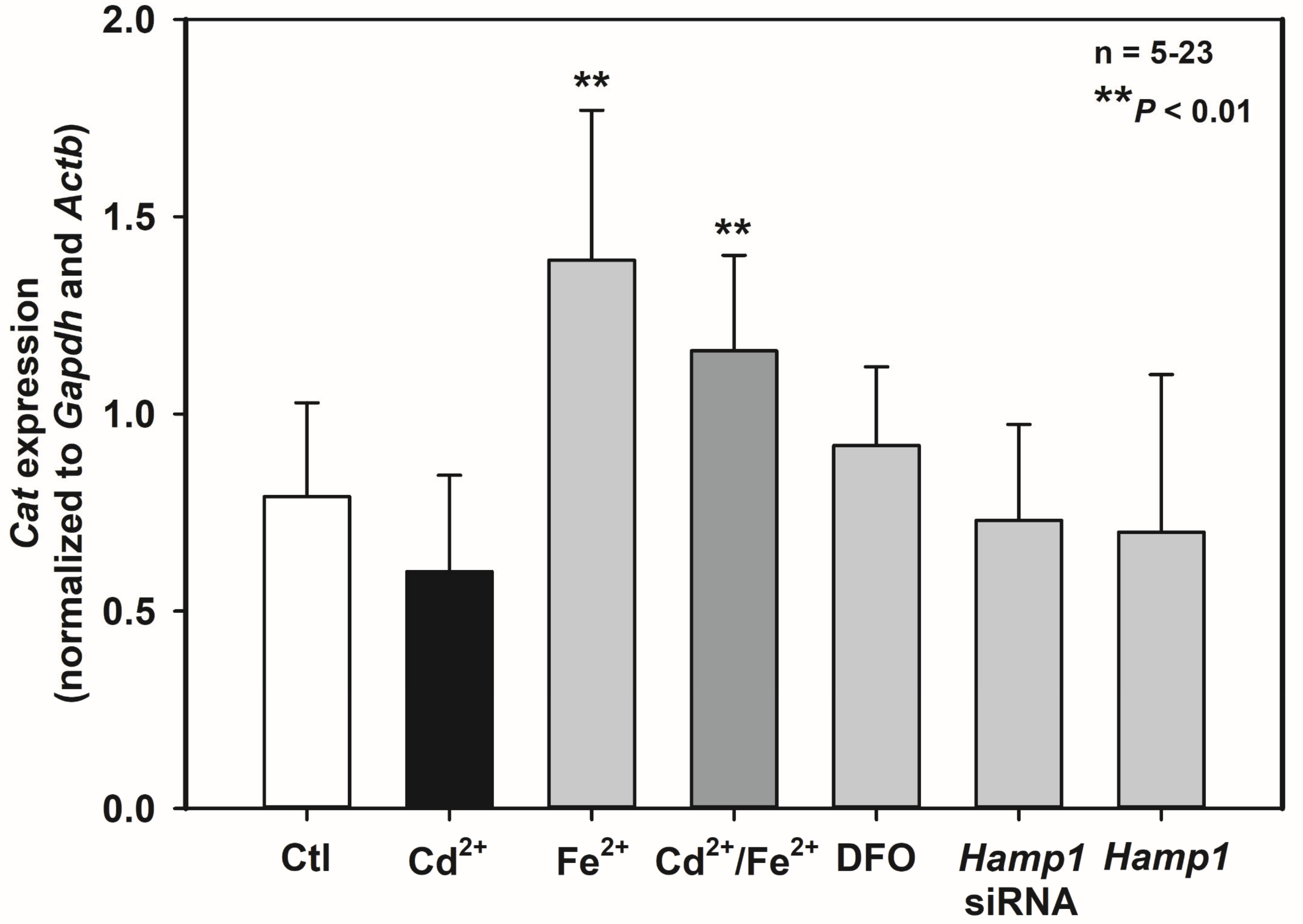

Suppl. Figure 4

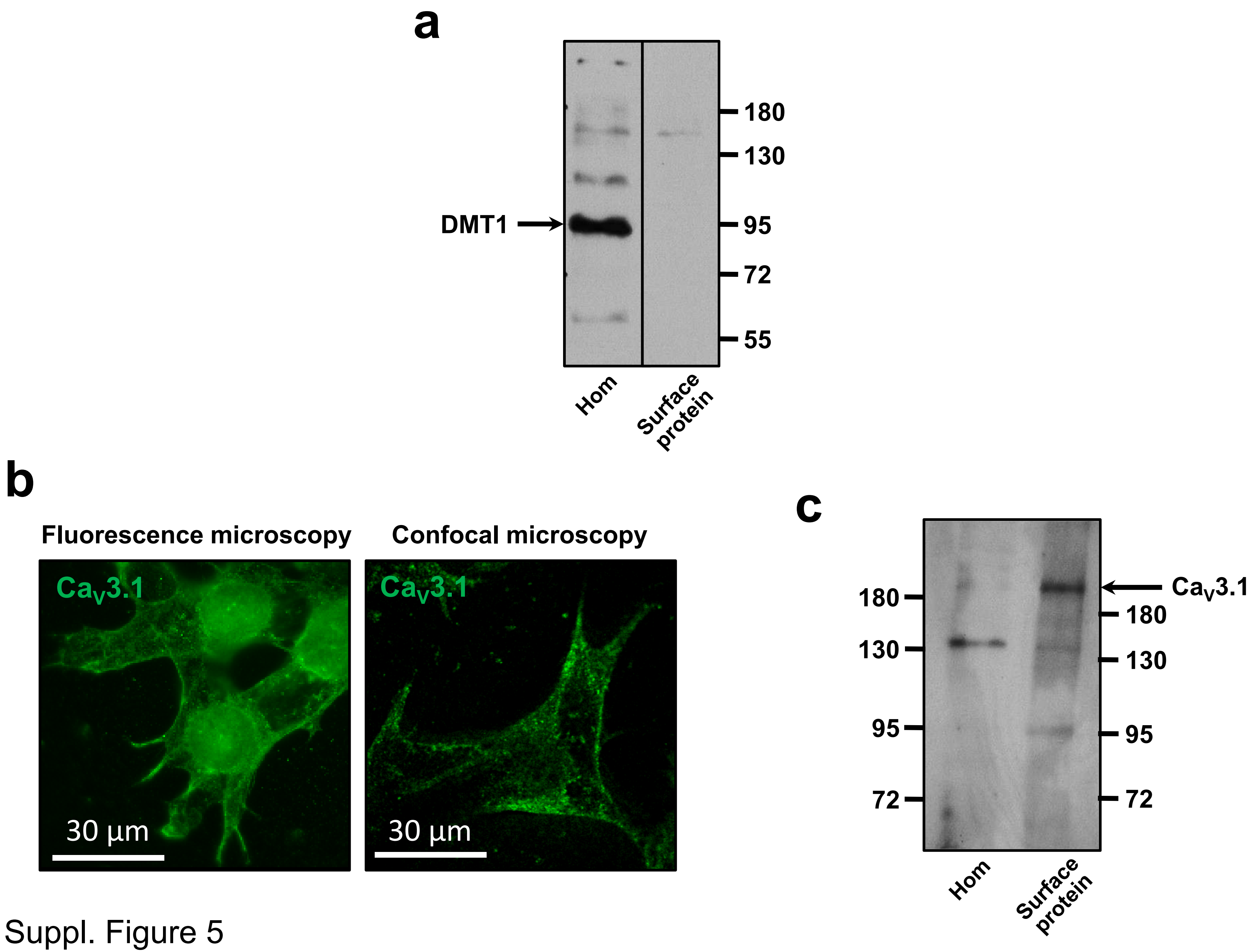

Suppl. Figure 5

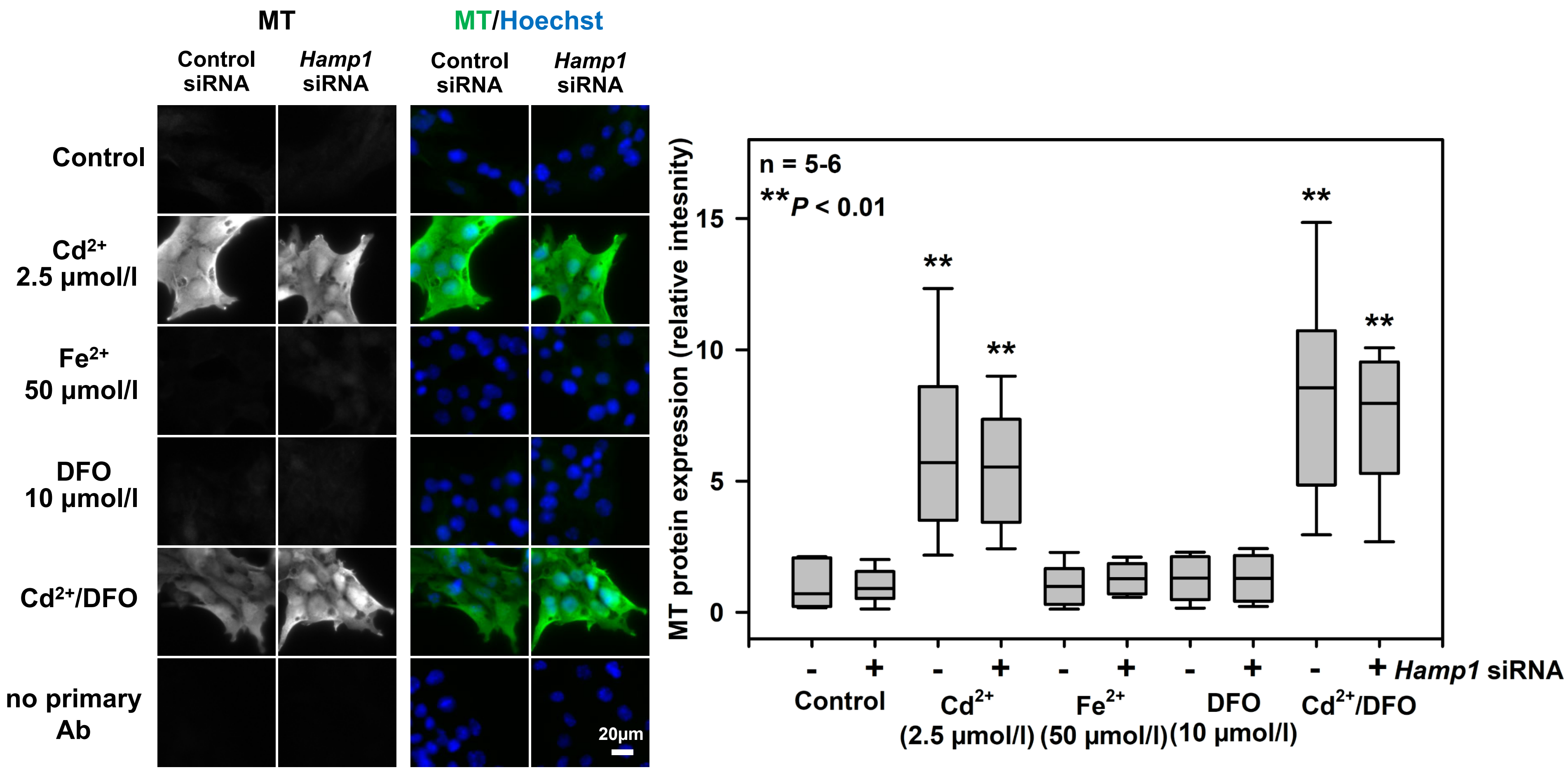

Suppl. Figure 6

**Method / Assay**	**Cell line**	**Seeding density**	**Plate format**	**Culture time**
Hamp1 RT-PCR/qPCR	$mIMCD_3$	1.0 x 10^5 / well	6-well	48 h
Hamp1 overexpression *Hamp1* qPCR	$mIMCD_3$	8.0 x 10^4 / well	12-well	24 h
Hamp1 qPCR	mCCD(cl.1)	1.25 x 10^5 / well	12-well	24h
Hamp1 RT-PCR/qPCR	WKPT-0293 Cl.2	2.5 x 10^5 / well	6-well	48 h
Cat qPCR	$mIMCD_3$	2.0 x 10^5 / well	6-well	24 h
Hamp1 overexpression *Cat* qPCR	$mIMCD_3$	8.0 x 10^4 / well	12-well	24 h
Catalase Assay	$mIMCD_3$	1.2 x 10^5 / well	12-well	24 h
Catalase Assay	$mIMCD_3$	2.0 x 10^5 / well	6-well	24 h
Catalase Assay	mCCD(cl.1)	1.0 x 10^5 / well	12-well	24h
Catalase Assay	WKPT-0293 Cl.2	3.5 x 10^5 / well	6-well	24 h
MTT	$mIMCD_3$	1.0 x 10^3 / well	24-well	72 h
MTT	$mIMCD_3$	3.0 x 10^4 / well	24-well	24 h
MTT	$mIMCD_3$	1.5 x 10^4 / well	48-well	24 h
Hamp1 overexpression MTT	$mIMCD_3$	4.0 x 10^4 / well	24-well	24 h
MTT	mCCD(cl.1)	2.5 x 10^4 / well	24-well	24h
Trypan Blue	$mIMCD_3$	2.5 x 10^4 / well	24-well	24 h
APOSTRAND™ ELISA Apoptosis Assay	$mIMCD_3$	1.0 x 10^3 / well	96-well	24 h
CellROX™ Green	$mIMCD_3$	5.0 x 10^3 / well	24-well	72 h
Hamp1 overexpression CellROX™ Green	$mIMCD_3$	4.0 x 10^4 / well	24-well	24 h
PARP immunoblotting	$mIMCD_3$	2.8 x 10^5 / well	6-well	24h

Suppl. Table 1: Seeding density, multiwell culture plate formats, and culture time before treatment for the methods/assays used in rat proximal tubule (WKPT-0293 Cl.2), mouse cortical (mCCD(cl.1) and inner medullary collecting duct ($mIMCD_3$) cell lines.

**Cell line**	**Relative mRNA** (normalized to *Gapdh* and *Actb*)	**Relative Protein** (O.D. normalized to β-actin)	**Enzymatic activity** (U/ml/µg protein)
WKPT-0293 Cl.2	0.46 ± 0.15	125.9 ± 41.9	30.3 ± 17.3
mIMCD3	0.18 ± 0.08	56.5 ± 16.7	7.3 ± 3.5
mCCD(cl.1)	n.d.	n.d.	5.3 ± 0.9
n	3	5	10-11

mRNA expression was determined by qPCR, protein expression by immunoblotting and enzymatic activity by fluorimetry (see Methods). Means ± SD are shown. n.d. = not determined

Suppl. Table 2: Catalase expression and activity in proximal tubule (WKPT-0293 Cl.2) and inner medullary collecting duct (mIMCD3) cell lines.
